# Relationship between surgeon volume and outcomes: a systematic review of systematic reviews

**DOI:** 10.1186/s13643-016-0376-4

**Published:** 2016-11-29

**Authors:** Johannes Morche, Tim Mathes, Dawid Pieper

**Affiliations:** 1 Institute of Health Economics and Clinical Epidemiology, The University Hospital of Cologne (AöR), Gleueler Str. 176-178, 50935 Cologne, Germany; 2 Faculty of Health, School of Medicine, Institute for Research in Operative Medicine, Witten/Herdecke University, Ostmerheimer Str. 200, Building 38, 51109 Cologne, Germany

**Keywords:** Systematic review of systematic reviews, Volume-outcome, Surgeon volume, Clinical outcome, Quality assurance, Patient safety

## Abstract

**Background:**

The surgeon volume-outcome relationship has been discussed for many years and its existence or nonexistence is of importance for various reasons. A lot of empirical work has been published on it. We aimed to summarize systematic reviews in order to present current evidence.

**Methods:**

Medline, Embase, Cochrane database of systematic reviews (CDSR), and health technology assessment websites were searched up to October 2015 for systematic reviews on the surgeon volume-outcome relationship. Reviews were critically appraised, and results were extracted and synthesized by type of surgical procedure/condition.

**Results:**

Thirty-two reviews reporting on 15 surgical procedures/conditions were included. Methodological quality of included systematic reviews assessed with the assessment of multiple systematic reviews (AMSTAR) was generally moderate to high albeit included literature partly neglected considering methodological issues specific to volume-outcome relationship. Most reviews tend to support the presence of a surgeon volume-outcome relationship. This is most clear-cut in colorectal cancer, bariatric surgery, and breast cancer where reviews of high quality show large effects.

**Conclusions:**

When taking into account its limitations, this overview can serve as an informational basis for decision makers. Our results seem to support a positive volume-outcome relationship for most procedures/conditions. However, forthcoming reviews should pay more attention to methodology specific to volume-outcome relationship. Due to the lack of information, any numerical recommendations for minimum volume thresholds are not possible. Further research is needed for this issue.

**Electronic supplementary material:**

The online version of this article (doi:10.1186/s13643-016-0376-4) contains supplementary material, which is available to authorized users.

## Background

In particular, in surgical disciplines, lots of studies have been published on the volume-outcome relationship since Luft et al. [1, 2] explained the theory of it. Mortality and survival have been explored most in this debate. Many different primary studies as well as systematic reviews indicate a positive relationship between hospital as well as surgeon volume and clinical outcomes for different surgical procedures [3–5]. It has been suggested that surgeon volume is more important than hospital volume for procedures with a shorter length of stay and specific intraoperative processes and skills (e.g., carotid endarterectomy) whereas hospital volume is suggested to be more important for those procedures which implicate longer lengths of stay and a major need for hospital-based services such as intensive or respiratory care (e.g., lung resection) [5].

The existence or nonexistence of surgeon volume-outcome relationship is important for different issues. It can be of importance for the methodological refinement of clinical studies on surgical innovations. The evaluation of innovations vs. established procedures can lead to biased results in terms of the comparison of the effects of the different procedures. These trials might overestimate effects for established procedures in comparison to innovations as surgeons are more familiar in performing these surgeries. Therefore, such trials might lead to better outcomes for established procedures only due to its longer existence and not due to the procedure itself [6]. Additionally, only few multicenter trials report about provider effects due to variation in expertise. Low-volume and high-volume providers are often included in the same trials which might cause misleading conclusions [7]. Moreover, it is also important to know whether high-volume surgeons (HVS) perform better in order to provide patients with a good medical treatment. A sound knowledge about surgeon volume-outcome relationship might have important implications for designing training for surgeons. Furthermore, minimum volume thresholds for surgeons might come into force. There already exist recommendations by the Expert Panel on Weight Loss Surgery [8] for bariatric surgery, and an international expert panel defined appropriate and inappropriate surgeon volumes for a variety of gastric procedures [9].

Many systematic reviews have been published on this topic, so that it becomes more and more difficult to deal with the huge amount of literature. Therefore, the specific scope of this paper is to provide an overview of all the systematic reviews and to perform a synthesis of the evidence on the surgeon volume-outcome relationship. We analyze if the clinical outcomes of patients undergoing any kind of surgery will be favorable if they are operated by HVS in comparison to low-volume surgeons. The synthesis is based on a thorough evaluation of the quality of the included reviews and their results in different surgical procedures/conditions.

## Methods

This systematic review of systematic reviews was undertaken in particular according to the methods prescribed in the chapter on overviews in the Cochrane Handbook for Systematic Reviews of Interventions [10] and is reported according to the Preferred Reporting Items for Systematic Reviews and Meta-analyses (PRISMA) [11] (see Additional file 1). There was no formal protocol for our work. However, being part of a master thesis, a short project proposal was prepared. Therein, it was specified a priori to follow basically the same methods as in the previous analysis of our research group on hospital volume [12].

### Literature search strategy

We performed a systematic literature search to identify all published systematic reviews on the association between surgeon volume and clinical outcomes. Medline (via Pubmed), Embase (via Embase), and Cochrane database of systematic reviews (via Wiley Online Library) were searched (all search strategies can be found in Additional file 2). Reference lists of relevant articles were hand-searched to identify additional articles not retrieved by our search strategy. Furthermore, we inspected websites of health technology assessment organizations that were members of INAHTA, HTAi, or EUnetHTA in October 2015 to identify reports not indexed in bibliographic databases (Additional file 3). All searches were done without time restriction in October 2015.

### Study selection

In consideration for this review, the following inclusion criteria were applied to each systematic review: review of primary studies derived by a systematic literature search, any kind of critical appraisal of included studies, addressing the relationship between surgeon volume and clinical outcomes in surgery/surgical procedures, and written in English or German. Articles dealing solely with the relationship between specialization or hospital volume and clinical outcomes were excluded. Systematic reviews investigating the relationship between both hospital volume and surgeon volume were included, if results for surgeon volume were reported separately or could be derived from text.

All titles and abstracts were screened independently by two members of the research team. The full texts of potentially eligible articles were obtained. Two reviewers assessed the eligibility of the full texts against the review inclusion criteria. Any disagreements were resolved by discussion.

### Data collection

Data were extracted by one reviewer into structured summary tables and checked for accuracy by a second reviewer. Any disagreements were discussed until consensus was reached. For each systematic review, characteristics were extracted on the surgical procedure/condition, inclusion and exclusion criteria for primary studies, search period, and number of included studies. As some systematic reviews included studies other than on surgeon volume (e.g., hospital volume), we quoted additionally the number of included studies reporting on the relationship between surgeon volume and outcomes. Results were extracted according to the type of evidence synthesis. In the case of narrative synthesis, results were abstracted by modified vote counting [13]. This contained data on comparisons showing HVS performing better (irrespective of statistical significance), median effect size (range) across all comparisons, comparisons showing statistically significant effects in favor of HVS, and total number of comparisons. This method has been suggested for presenting results of qualitative synthesis, overcoming problems arising when simple vote counting is used by relying either on the number of comparisons with a positive direction of effect or the number of comparisons reaching statistical significance. Studies with low statistical power could be misleading in interpretation of overall effects in synthesis [10, 14, 15]. If multiple comparisons were given, in terms of more than two volume categories, we relied on the effect sizes of the highest volume surgeons opposed to the lowest volume surgeons. For example, if a study used four volume categories and defined the highest volume category as the reference, authors might report three different odds ratios (OR) (or any other effect measure) when categories were opposed to HVS (the lowest vs. HVS, low vs. HVS, medium vs. HVS). In this case, we relied on the OR corresponding to the lowest vs. HVS. For all meta-analyses, we extracted pooled effect sizes, confidence intervals, types of effect modelling, measures of statistical heterogeneity (*I* ^2^), and the numbers of comparisons in addition to the data needed for modified vote counting. Low-volume surgeons were used as reference category within this overview so that effect measures for mortality will be smaller than one and effect measures for survival will be bigger than one if HVS perform better than low-volume surgeons. If included systematic reviews reported effect measures differently and used HVS as reference category, effect measures were converted so that results can be interpreted consistently across different reviews. We referred to comparisons instead of the number of studies, because some studies included more than one comparison used in meta-analysis. We assumed only observational studies to be included in the systematic reviews. Confounding is known to be a major problem in this study design [16, 17], so we extracted data irrespective of the type of synthesis on case-mix adjustments by means of variables that were adjusted for in each study for a given outcome and condition where at least two studies were synthesized. Data on case-mix adjustments were not extracted where only one study was available. We reported results based on surgical procedure/condition. Within the result section of a specific procedure/condition, we state whether a procedure (e.g., Norwood procedure) or a condition (e.g., breast cancer) was considered. We calculated the “corrected covered area” (CCA) in order to investigate the overlap of primary studies included in different systematic reviews for the same procedure/condition [18]. The first occurrence of a primary publication is defined as the index publication. The CCA divides the frequency of repeated occurrences of the index publication in other reviews by the product of index publications and reviews, reduced by the number of index publications. It is used as it allows a classification into slight (0–5%), moderate (6–10%), high (11–15%), and very high (>15%) overlap for different surgical procedures/conditions.

### Assessment of review quality

Methodological quality of the eligible systematic reviews was undertaken independently by two reviewers. Any disagreements were resolved by discussion. We used the “assessment of multiple systematic reviews” (AMSTAR) [19] which includes 11 items to judge the quality of each systematic review (Additional file 4). AMSTAR was found to be a reliable and valid measurement tool to assess the methodological quality of systematic reviews [20, 21], and it seems that all items can generally be applied to systematic reviews of non-randomized studies [22]. We added a supplemental question on reporting of dealing with multiple comparisons in primary studies. Some studies might have calculated effect sizes using more than two volume categories (e.g., high, middle, low). In these cases, authors should clearly state which comparison was chosen (e.g., the highest volume group opposed to the lowest volume group), as this might have an influence on results. We judged this to be not applicable where results of all comparisons were reported in case of narrative evidence synthesis. The requirement for the item “conflict of interest” was changed in comparison to the description of the authors of AMSTAR. The authors demand that “potential sources of support should be clearly acknowledged in both the systematic review and the included studies” [19]. We considered the item as being fulfilled if potential sources of support in the systematic review were clearly acknowledged.

### Evidence synthesis

A meta-analysis of systematic reviews is difficult as some of the primary studies will usually be included in more than one review. Pooling results would give too much statistical power to multiple included primary studies [23]. Thus, we performed a qualitative evidence synthesis by assessing the surgeon volume-outcome relationship on the body of evidence (taking overlaps of primary studies into account), quality of systematic reviews, consistency of findings, and up-to-dateness of the body of evidence. We rated the relationship on an ordinal scale with tendency/trend (+), moderate (++), strong (+++), unclear (?), and no relationship (−). We already applied this approach satisfactorily in our earlier systematic review of systematic reviews on the hospital volume-outcome relationship [12].

## Results

**Fig. 1:**
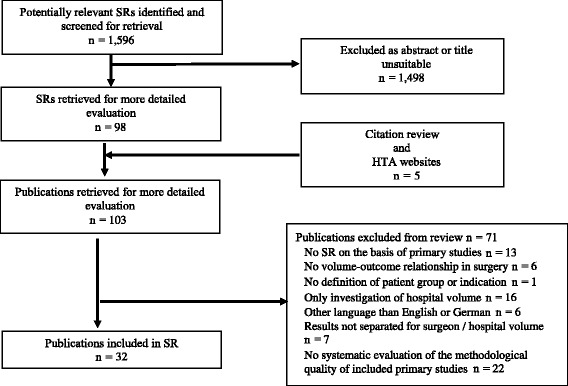
Flow chart

From 1596 abstracts initially identified, 98 were retrieved for more detailed evaluation. Five additional studies were identified by citation review and hand-searching HTA websites. In total, 103 publications were screened in full-text of which 71 had to be excluded (see Additional file 5 for the list of excluded reviews), leaving 32 systematic reviews [3, 24–54] suitable for inclusion (see Fig. 1, based on Additional file 1 [11]).

Twenty-six of the included reviews focus on one specific procedure/condition. These reviews examine the surgeon volume-outcome relationship for 15 different procedures/conditions. Six systematic reviews focus on colorectal cancer [24, 25, 35, 36, 43, 48], three on bariatric surgery [37, 41, 54], two on abdominal aortic aneurysm (AAA) [50, 53], two on esophageal cancer [26, 52], two on radical prostatectomy [47, 51], and two on total knee arthroplasty [38, 45]. Single systematic reviews report on breast cancer [30], on coronary artery bypass graft (CABG) [44], on cystectomy [32], on head and neck cancer [28], on lung cancer [49], on Norwood procedure [42], on pancreatic surgery [31], on percutaneous coronary intervention (PCI) [46], and on trauma [27]. All of these reviews focus mainly on adults except for the review about Norwood procedure which only analyzes neonates. Six additional reviews include information about several different surgical procedures/conditions [3, 29, 33, 34, 39, 40].

It was decided to use these general reviews in a supplementary manner. Where appropriate (e.g., in the absence of other meaningful up-to-date reviews) results of these reviews are partly discussed in the full sections below. Three of these reviews are the first ones that dealt with the volume-outcome relationship in surgery [29, 33, 34]. Thus, they are likely not to present the current state of evidence. See Additional file 6 for the characteristics of the included systematic reviews—condition/procedure analyzed, inclusion criteria for primary studies, relevant/total number of primary studies included—and Additional file 7 for a detailed description of empirical results. Based on the reporting within the systematic reviews, the vast majority of primary studies is based on data from the USA. Other studies used data from Canada, Europe (mostly UK, following Scandinavia), Australia, East Asia (Taiwan and Japan), and Brazil. As a number of reviews did not present these characteristics, there might also be studies using data from other regions/countries.

### Review quality

The methodological quality of included reviews (Table 1) was generally moderate to high, although some single reviews could even be judged as excellent and some other reviews had major methodological flaws. The most common methodological weakness was the lack of a list of studies (included and excluded), which was mostly due to a missing list of excluded studies. Two thirds of the reviews abstained from listing all included and excluded studies. Assessment of included primary studies differed among the systematic reviews. Approximately half of the reviews did not precisely report which criteria they used for assessing the methodological quality of primary studies or they did not present their results. In this case, we assessed the item on “critical appraisal” by AMSTAR as being not fulfilled. Most of the other reviews used a modified version of an existing tool (e.g., Newcastle-Ottawa Scale), referred to the STROBE statement, or used a newly arranged combination of criteria. Nevertheless, all of the reviews conducted some kind of critical appraisal as preconditioned for the inclusion into the overview. Approximately one out of four reviews did not appropriately consider methodological rigor and scientific quality of the primary studies in formulating conclusions. Moreover, one half of the reviews did not clearly describe that study selection as well as data extraction was conducted by two reviewers independently. One review fulfilled all quality criteria [31] and another review fulfilled all applicable criteria [42].

**Table 1:** Critical appraisal

Procedure/ condition	Study	A priori design	Two reviewers	Literature search	Status of publication	List of studies	Study characteristics	Critical appraisal	Conclusions	Combining findings	Publication bias	Conflict of interest	Multiple comparisons
Colorectal cancer	Archampong et al. 2012 [24]	+	+	+	+	+	+	+	+	+	+	+	?
	Archampong et al. 2010 [25]	+	+	+	+	−	+	+	+	+	+	+	+
	Van Gijn et al. 2010 [48]	+	+	+	+	−	+	−	−	+	+	+	+
	Salz et al. 2008 [43]	+	−	−	+	−	+	−	+	□	□	−	+
	Iversen et al. 2007 (short-term) [35]	+	?	+	+	−	−	?	+	+	+	−	−
	Iversen et al. 2007 (long-term) [36]	+	?	+	+	−	−	?	+	+	+	−	−
Bariatric surgery	Zevin et al. 2012 [54]	+	−	+	−	−	+	+	−	□	□	+	□
	Padwal et al. 2011 [41]	+	+	+	+	−	+	−	−	□	□	+	□
	Klarenbach et al. 2010 [37]	+	+	+	+	−	+	−	−	□	□	□	□
AAA	Young et al. 2007 [53]	+	−	−	−	+	−	−	−	+	−	+	+
	Wilt et al. 2006 [50]	+	−	−	−	+	+	−	+	□	□	+	+
Esophageal cancer	Brusselaers et al. 2014 [26]	+	−	+	+	−	+	−	−	+	+	+	+
	Wouters et al. 2012 [52]	+	+	−	+	−	+	+	+	+	+	+	?
Radical prostatectomy	Trinh et al. 2013 [47]	+	?	+	−	−	+	−	+	□	□	+	□
	Wilt et al. 2008 [51]	+	?	+	+	+	+	+	+	?	−	−	+
Total knee arthroplasty	Lau et al. 2012 [38]	+	+	+	+	+	+	?	?	□	□	+	+
	Stengel et al. 2004 [45]	+	?	−	+	−	+	−	+	□	□	+	+
Breast cancer	Gooiker et al. 2010 [30]	+	+	+	+	−	+	+	+	+	+	+	+
CABG	Sepehripour et al. 2013 [44]	+	?	−	−	−	+	+	+	□	□	+	□
Cystectomy	Goossens-Laan et al. 2011 [32]	+	+	+	?	−	+	+	+	+	+	+	+
Head and neck cancer	Eskander et al. 2014 [28]	+	+	+	+	−	+	+	+	+	□	+	+
Lung cancer	Van Meyenfeldt et al. 2012 [49]	+	+	+	+	+	+	−	+	+	+	+	?
Norwood procedure	Pieper et al. 2014 [42]	+	+	+	+	+	+	+	+	□	□	+	□
Pancreatic surgery	Gooiker et al. 2011 [31]	+	+	+	+	+	+	+	+	+	+	+	+
PCI	Strom et al. 2014 [46]	+	+	?	−	−	?	−	+	+	+	+	+
Trauma	Caputo et al. 2014 [27]	+	−	+	+	−	+	−	+	□	□	+	□
Several	Gruen et al. 2009 [3]	+	+	+	+	−	+	+	+	□	□	−	−
	Miyata et al. 2007 [40]	+	?	+	+	−	+	+	−	□	□	−	□
	Gandjour et al. 2003 [29]	+	?	+	+	−	−	+	−	□	−	−	−
	Halm et al. 2002 [33]	+	+	−	+	−	+	−	+	□	□	−	+
	Hillner et al. 2000 [34]	+	?	−	−	−	−	−	+	□	□	−	−
Several in pediatric surgery	McAteer et al. 2013 [39]	+	?	+	+	−	+	+	+	□	□	+	−

+ yes, − no, ? cannot answer, □ not applicable

*Abbreviations*: *AAA* Abdominal Aortic Aneurysm, *CABG* coronary artery bypass graft, *PCI* percutaneous coronary intervention

### Colorectal cancer

There are six systematic reviews which evaluate the relationship between surgeon volume and outcomes for the condition colorectal cancer [24, 25, 35, 36, 43, 48]. The reviews included 22 [24], 15 [35], 11 [25, 36, 43], and seven [48] primary studies. In total, the reviews included 40 different primary studies. The most recent published review by Archampong et al. [24] included all primary studies that were included in their earlier publication [25] and eleven additional primary studies. Both reviews (short-term and long-term outcomes) by Iversen et al. [35, 36] used the same methodological framework, and seven primary studies were included in both reviews.

Thirty-day or postoperative mortality was investigated within five reviews [24, 25, 35, 43, 48]. The OR regardless of the location of the cancer was 0.77 (95% CI 0.66–0.91; *I* ^2^ = 56%) [24]. Results for the different cancer locations were heterogeneous. All pooled ORs were significant for colon cancer with 0.75 (95% CI 0.62–0.92; *I* ^2^ = 71%) [24], 0.50 (95% CI 0.39–0.64; *I* ^2^ = 85.4%) [35], and 0.82 (95% CI 0.68–0.99) [48]. The ORs for colorectal cancer were 0.82 (95% CI 0.54–1.24; *I* ^2^ = 68.0%) [35] and 0.67 (95% CI 0.53–0.84; *I* ^2^ = 45.6%) [48]. The ORs for rectal cancer were not significant being 0.72 (95% CI 0.44–1.17; *I* ^2^ = 34.3%) [35], 0.86 (95% CI 0.62–1.19; *I* ^2^ = 0%) [24], and 0.79 (95% CI 0.59–1.06; *I* ^2^ = 0%) [25].

Five reviews investigated overall or cancer-specific survival [24, 25, 36, 43, 48]. The hazard ratio (HR) for overall 5-year survival was 1.14 (95% CI 1.08–1.20; *I* ^2^ = 26%) [24]. The result for the 5-year disease-specific survival was not significant with a HR of 1.06 (95% CI 0.87–1.30; *I* ^2^ = 0%) [24]. All of the primary studies for colon cancer demonstrated significant results favoring HVS concerning overall survival [24, 36, 48]. The result for the 5-year disease-specific survival showed no favorable effect for HVS [24]. All pooled results for colorectal cancer favored HVS [24, 36, 48], and two of these pooled results were statistically significant [24, 48]. One [25] of three [24, 25, 36] pooled results showed statistically significant longer survival for HVS for rectal cancer. The HR for the 5-year disease-specific survival was not significant [24].

Abdominoperineal excision of the rectum for rectal cancer was investigated in two reviews. The ORs for abdominoperineal excision including and excluding rectosigmoid cancer were 0.58 (95% CI 0.45–0.76; *I* ^2^ = 67%) and 0.51 (95% CI 0.28–0.93; *I* ^2^ = 85%;), respectively [25]. The result of the other review confirms a significantly lower rate of abdominoperineal excision for HVS by one primary study [24].

The overall OR for anastomotic leak was 0.64 (95% CI 0.40–1.02; *I* ^2^ = 0%) [24]. All analyses for specific locations favor HVS without entailing significant results [24, 25].

Three out of four primary studies showed a significant lower local recurrence rate for HVS [43]. Another review confirms this trend with a significant result [25]. Additionally, there was a significantly lower rate of permanent stoma for HVS [24, 36]. The CCA of 23.59% indicates a very high overlap of primary studies between the different systematic reviews.

### Bariatric surgery

There are three systematic reviews on bariatric surgery for the condition obesity, and all of them show positive volume-outcome relationship [37, 41, 54]. Two of these reviews were conducted by the same researchers with a similar methodology [37, 41]. Therefore, six of the seven primary studies which were included in the former publication [37] were also included in the later one [41]. In total, the reviews included 16 different primary studies.

The reviews included 13 [54], eight [41], and seven primary studies [37], and all of them refrained from pooling results in a quantitative way. They show that surgeon volume and mortality are related inversely. In six out of eight primary studies included by one review, there was a statistically significant lower mortality when operated by HVS [54]. The other two reviews [37, 41] included three primary studies which were not included in the most up-to-date review [54]. Nevertheless, the results do not differ essentially between each other. Five of six [41] and three of four [37] included primary studies showed significant results, and simultaneously all of the primary studies showed lower mortality rates for HVS.

Similar to the results regarding mortality, the reviews show that higher surgeon volume is related to lower rates of complications, surgical sequelae, and adverse outcomes such as death, non-routine hospital transfer, or venous thromboembolism. These outcomes were analyzed in six primary studies included in one review, and all of them showed significantly less complications or adverse outcomes for patients treated by HVS [54]. This trend is supported by the results for surgical sequelae of the other reviews [37, 41]. The CCA of 37.50% indicates a very high overlap of primary studies between the different systematic reviews.

### Abdominal aortic aneurysm

Both systematic reviews investigating the condition unruptured/elective AAA show positive volume-outcome relationship [50, 53]. In total, these reviews included 14 different primary studies.

Mortality was analyzed in 14 primary studies. All of them were included in one of the reviews and the pooled OR of six eligible studies was 0.56 (95% CI 0.54–0.57; *I* ^2^ = 23.7%) [53] indicating that surgeons with more than 13 annual surgeries perform better than their colleagues with less annual surgeries. The other systematic review [50] included four primary studies but all of them were also included by the more recent one [53]. The authors of the older review refrained from pooling the results of the primary studies in a quantitative way but they stated that all four included primary studies demonstrate significantly lower in-hospital mortality for patients treated by HVS [50]. The CCA of 28.57% indicates a very high overlap of primary studies between the different systematic reviews.

### Esophageal cancer

There are two systematic reviews for the condition esophageal cancer [26, 52]. In total, these reviews included 14 different primary studies. The authors of one of the reviews only considered three high-quality studies for their meta-analysis, and pooling yielded an OR of 0.87 (95% CI 0.36–1.14; *I* ^2^ = 75%) [52]. Additionally, one of the reviews analyzing more than one procedure/condition included six primary studies investigating the relation between surgeon volume and short-term mortality with all of them showing significantly lower mortality rates for HVS [3]. The HRs for long-term survival were 1.14 (95% CI 0.98–1.35; *I* ^2^ = 0%; *n* = 3) [26] and 1.16 (95% CI 0.94–1.45; *I* ^2^ = 48%; *n* = 2) [52]. The CCA of 14.29% indicates a high overlap of primary studies between the different systematic reviews.

### Radical prostatectomy

There are two systematic reviews for the procedure radical prostatectomy [47, 51]. These two reviews included 33 [47] and ten [51] primary studies. In total, they included 35 different primary studies. The results were separated within one of the reviews depending on the surgical technique (open vs. laparoscopic) [47]. One primary study included into this review showed a significantly lower postoperative mortality for HVS whereas another primary study did not demonstrate a significant result regarding 30-day mortality [47]. Likewise, the pooled analysis of two primary studies did not demonstrate a significant decrease in surgery-related mortality with more operations [51]. One of the reviews analyzed several patient-related outcomes, and most primary studies indicated significant lower rates of long-term incontinence, complications, anastomotic strictures, and positive surgical margins as well as a significant lower risk of additional therapies for patients treated by HVS [47]. The results for the two first-mentioned outcomes are supported by the other review with significant results [51]. The CCA of 22.86% indicates a very high overlap of primary studies between the different systematic reviews.

### Total knee arthroplasty

There are two systematic reviews for the procedure total knee arthroplasty [38, 45]. In total, these reviews included 14 different primary studies. All of the three primary studies investigating 90-day mortality and included in one of the reviews indicated a lower mortality rate for patients treated by HVS albeit the result of one primary study was not reported completely precise. None of the studies entailed significant results [38]. Similarly, the primary study included in the other review indicated a lower 90-day mortality rate without entailing statistically significant results and the same is true for the two studies investigating in-hospital mortality [45]. Another primary study indicated lower in-hospital mortality for HVS but significance was not reported [38]. One of the systematic reviews investigating several surgical procedures/conditions found significantly lower mortality rates for primary as well as for revision knee replacement. Both outcomes were analyzed in one primary study [29]. Results for other outcomes were heterogeneous. One of the reviews did not entail significant results regarding clinical outcomes [45] but the other review [38] indicates significantly better outcomes for HVS regarding pneumonia, the inability to flex the knee to 90 °, the inability to achieve full extension at 2 years postoperation, and for WOMAC score. For most other outcomes results indicate better effects for HVS without being statistically significant [38]. The CCA of 15.38% indicates a very high overlap of primary studies between the different systematic reviews.

### Breast cancer

The systematic review for the condition breast cancer included seven primary studies, and all of them show results in favor of HVS regarding survival [30]. Six of the seven primary studies included significant results. The pooled effect size of studies with hazard ratios was HR 1.22 (95% CI 1.08–1.39; *I* ^2^ = 59%) and with relative risks (RR) was RR 1.18 (95% CI 1.10–1.25; *I* ^2^ = 0%) [30].

### Coronary artery bypass graft

There is one systematic review based on three primary studies for the procedure off-pump CABG [44, 46]. One out of two included primary studies favored HVS for in-hospital mortality without showing significant results [44]. The third primary study showed statistically significant lower mortality rates for patients treated by HVS for three different points in time. The authors of the review refrained from defining these points in time [44]. Two systematic reviews dealing with several procedures/conditions investigated mortality for CABG. All three primary studies included in one review showed significant lower mortality rates for patients treated by HVS [33] whereas the other review included one primary study showing a non-significant lower mortality rate for HVS [29].

### Cystectomy for bladder cancer

There is one systematic review for the procedure radical cystectomy for bladder cancer based on three primary studies [32]. The pooled OR for postoperative mortality was 0.58 (95% CI 0.46–0.73; *I* ^2^ = 50%). The primary study analyzing the relation between surgeon volume and survival also favored HVS but without showing significant results [32].

### Head and neck cancer

There is one systematic review for the condition head and neck cancer based on nine primary studies [28]. The included studies focused on larynx surgery, on neck dissection, on oropharyngeal surgery, and on surgery of the oral cavity.

Long-term survival and long-term mortality (three or five years) were only examined for surgery of the oral cavity. The 3-year overall survival for surgery of the oral cavity with flap or predicted reconstruction as well as the 5-year overall survival for oral cavity resection were significantly longer for patients treated by HVS. The analysis of long-term mortality showed a HR of 0.77 (95% CI 0.64–0.92; *I* ^2^ = 0%). In-hospital mortality was examined for larynx and oropharyngeal surgery. For both surgeries one out of two primary studies favored HVS without entailing significant results [28]. One primary study showed significantly lower rates of regional recurrence after 9 months of follow-up and harvested number of lymph nodes from neck dissection for neck dissection [28].

### Lung cancer

There is one systematic review for the condition lung cancer [49]. Both primary studies included in this review showed a significantly lower postoperative mortality for patients treated by HVS. However, the pooled result was not significant with an OR of 0.67 (95% CI 0.42–1.08; *I* ^2^ = 66%). Two primary studies included by two other systematic reviews which analyzed more than one procedure/condition showed lower rates of 30-day mortality [33] and of mortality (not defined) [29] for HVS without including significant results.

### Norwood procedure

There is one systematic review for Norwood procedure based on four primary studies [42]. Two primary studies showed lower mortality for HVS albeit only the results of one study showed statistical significance. One study investigating survival also favored HVS without entailing significant results. Length of ventilation and time to first extubation were non-significantly shorter for HVS. The rate of renal failure was higher for HVS without entailing significant results [42].

### Pancreatic surgery

There is one systematic review for surgery on the condition pancreatic cancer based on three primary studies [31]. Moreover, there are four further systematic reviews dealing with several surgical procedures/conditions which also examined surgeon volume-outcome relationship for pancreatic surgery [3, 29, 33, 40]. The pooled OR for mortality was 0.46 (95% CI 0.17–1.26; *I* ^2^ = 94%) with high heterogeneity [31]. Another included study showed a significantly lower mortality for patients treated by HVS [31]. Five of eleven [3] and one out of two [33] primary studies demonstrated significantly lower short-term [3] or 30-day [33] mortality for patients treated by HVS. The same was shown for one out of two primary studies for long-term mortality [3].

### Percutaneous coronary intervention

There is one systematic review based on 21 primary studies for the procedure PCI. There was no significant relationship for in-hospital or 30-day mortality with an OR of 0.96 (95% CI 0.86–1.08; *I* ^2^ = 61.4%) [46]. Mortality was also investigated within two of the systematic reviews dealing with several procedures/conditions. One out of five primary studies showed significantly lower mortality rates for patients treated by HVS for coronary angioplasty [33] and five out of six primary studies included in another review favored HVS with two of them entailing significant results [29]. The pooled OR for major cardiac events was 0.62 (95% CI 0.40–0.97; *I* ^2^ = 96.6%) [46].

### Trauma

There is one systematic review for trauma injury patients based on four primary studies [27]. One out of these four primary studies yielded a lower in-hospital mortality rate for patients treated by HVS but the authors of the review did not report whether the results of the primary studies were significant or not.

### Evidence synthesis

The strongest associations were found for colorectal cancer, bariatric surgery, and breast cancer. For all three conditions/kinds of surgery the relationship between surgeon volume and outcomes was rated as moderate (++). The accomplishment of this rating is quite different for the three conditions/kinds of surgery. The body of evidence was largest for colorectal cancer with six systematic reviews based on 40 different primary studies and the most recent as well as methodologically best reviews clearly support a relationship between surgeon volume and outcomes [24, 25, 48]. For bariatric surgery, there are three main systematic reviews on the basis of two methodical approaches with good methodological quality and their results clearly support a relationship between surgeon volume and outcomes [37, 41, 54]. For breast cancer, on the other hand, there is only one main systematic review that clearly supports a surgeon volume-outcome relationship but its methodological quality is excellent and therefore results are trustworthy [30].

A tendency/trend of surgeon volume-outcome relationship was found for the following procedures/conditions: AAA, cystectomy, esophageal cancer, head and neck cancer, lung cancer, pancreatic surgery, radical prostatectomy, and total knee arthroplasty. Although both included systematic reviews analyzing AAA show a clear correlation between surgeon volume and outcomes, the relationship is rated as tendency/trend as the quality of the systematic reviews is not convincing [50, 53]. The body of evidence for cystectomy is limited with only three included primary studies but the systematic review is of high methodological quality, and the effect for mortality is large [32]. The same is true for head and neck cancer as all outcomes were analyzed only by one or two primary studies [28]. The respective systematic reviews for esophageal cancer [26, 52] and total knee arthroplasty [38, 45] included in this overview differ in their results regarding the extent of a relationship. The respective reviews that are more up-to-date indicate a stronger relationship than the older ones. For lung cancer, there is an overall relationship according to the results of the main systematic review [49] and the two reviews analyzing different procedures/conditions [29, 33] although these reviews only included four different primary studies in total. The relationship for pancreatic surgery is rated as tendency/trend due to the high statistical heterogeneity of the primary studies included and pooled within the systematic review [31]. The aggregate surgeon volume-outcome relationship for prostatectomy is also categorized as tendency/trend as results for many different patient-related outcomes significantly favor HVS but results were not consistent enough to justify a higher rating [47, 51].

For off-pump CABG the relationship between surgeon volume and outcomes is rated as unclear as the methodological quality of the review is flawed [44, 46]. It is rated as unclear for PCI as the pooled results for major adverse cardiac events are statistically very heterogeneous [46]. The surgeon volume-outcome relationship for trauma is also scored as unclear as the included primary studies are more than 10 years old and the review does not entail enough information to justify another rating [27]. The relationship for Norwood procedure receives the same classification as the body of evidence is not sufficient and results are heterogeneous for different outcomes [42]. Generally, overlapping of primary studies in different systematic reviews analyzing the same procedure/condition assessed by CCA was high to very high. Table 2 shows a summary assessment of the surgeon volume-outcome relationship for each procedure/condition as well as our own conclusions to the systematic reviews.

**Table 2:** Assessment of surgeon volume-outcomes relationship

Procedure/condition	Relationship	Study	End of search period	Reviewers’ conclusions
Colorectal cancer	++	Archampong et al. 2012 [24]	9/2011	Results are significantly better for HVS concerning mortality, overall survival, permanent stoma, and abdominoperineal excision; not significant for disease-specific survival and anastomotic leak
		Archampong et al. 2010 [25]	3/2010	Results are significantly better for HVS concerning overall survival, permanent stoma, abdominoperineal excision, and local recurrence; not significant for mortality and anastomotic leak
		Van Gijn et al. 2010 [48]	2/2010	Data clearly support a relation between surgeon volume and mortality as well as survival
		Salz et al. 2008 [43]	4/2007	No overall conclusions can be made due to heterogeneous study results and flaws in the methodological quality of the systematic review
		Iversen et al. 2007 (short-term) [35]	6/2004	Data indicate a relation between volume and short-term mortality for colon cancer but no statistically significant results for colorectal and rectal cancer
		Iversen et al. 2007 (long-term) [36]	6/2004	Data indicate a relation between volume and long-term survival for colon cancer but no statistically significant results for colorectal and rectal cancer
Bariatric surgery	++	Zevin et al. 2012 [54]	4/2011	Data clearly support the relationship between HVS and several patient-related outcomes including mortality
		Padwal et al. 2011 [41]	1/2011	Data clearly support the relationship between HVS and several patient-related outcomes including mortality
		Klarenbach et al. 2010 [37]	2/2009	Data clearly support the relationship between HVS and several patient-related outcomes including mortality
AAA	+	Young et al. 2007 [53]	NR	Pooled effect sizes for mortality were significant but findings should be interpreted with caution due to the quality of the data
		Wilt et al. 2006 [50]	10/2005	Data clearly support the relationship between HVS and in-hospital mortality
Esophageal cancer	+	Brusselaers et al. 2014 [26]	9/2013	Data indicate a relationship between volume and long-term survival
		Wouters et al. 2012 [52]	7/2010	No significant results for patient-related outcomes albeit data indicate a slight relationship between volume and mortality as well as survival
Radical prostatectomy	+	Trinh et al. 2013 [47]	12/2011	Data indicate a relation between volume and lots of different patient-related outcomes albeit there are no clear results for mortality
		Wilt et al. 2008 [51]	11/2007	Data indicate a relation between volume and different complications albeit the result for mortality is not significant
Total knee arthroplasty	+	Lau et al. 2012 [38]	12/2011	Data indicate a relation between volume and many different patient-related outcomes but results are significant only for about the half of the outcomes
		Stengel et al. 2004 [45]	NR	Data do not support a significant relationship between surgeon volume and patient-related outcomes albeit most outcomes are better for HVS
Breast cancer	++	Gooiker et al. 2010 [30]	2/2010	Data of this methodologically excellent review clearly support the relationship between HVS and survival
CABG	?	Sepehripour et al. 2013 [44]	7/2012	Data do not indicate a clear relation between volume and mortality or other patient-related outcomes and results should be interpreted with caution due to methodological shortcomings
Cystectomy	+	Goossens-Laan et al. 2011 [32]	9/2010	Data show a significant relation between volume and mortality whereas the result for survival is not significant and the body of evidence is limited
Head and neck cancer	+	Eskander et al. 2014 [28]	3/2013	Data indicate a relationship for volume and long-term mortality/survival for surgery of the oral cavity but no significant results for in-hospital mortality for larynx or oropharyngeal surgery
Lung cancer	+	Van Meyenfeldt et al. 2012 [49]	1/2011	Data show a relationship between surgeon volume and postoperative mortality
Norwood procedure	?	Pieper et al. 2014 [42]	3/2013	Data might indicate a slight relationship between volume and patient-related outcomes but the results are heterogeneous and predominantly non-significant
Pancreatic surgery	+	Gooiker et al. 2011 [31]	2/2010	Data indicate a relation between volume and postoperative mortality albeit studies are heterogeneous
PCI	?	Strom et al. 2014 [46]	9/2012	Data indicate a relation between volume and major adverse cardiac events but there is no relationship between volume and mortality and pooled results are very heterogeneous
Trauma	?	Caputo et al. 2014 [27]	6/2013	The review included only four primary studies which are more than 10 years old and it does not report on statistical significance

Authors’ assessment on the surgeon volume-outcome relationship is based on the body of evidence (taking overlaps of primary studies into account), quality of systematic reviews, consistency of findings and up-to-dateness of the body of evidence: + tendency/trend, ++ moderate, +++ strong, ? unclear, and − no relationship. *NR* not reported

## Discussion

This systematic review of systematic reviews provides an overview of the best current evidence for the surgeon volume-outcome relationship. Special emphasis was put on critical appraisal of included literature and special methodological aspects of dealing with multiple comparisons and case-mix adjustments. This has been criticized in the past [33, 55, 56], but was accounted for in some recently published reviews. Quality of included reviews was moderate to high with a tendency towards higher review quality in the recent past. This is in accordance with prior findings that indicated an increasing quality of reporting of meta-analyses with time [57].

Similarly to the results of our previous work about hospital volume-outcome relationship [12], there is a surgeon volume-outcome relationship for most procedures/conditions as well. Based on the included systematic reviews, this association tends to be stronger for hospital volume than for surgeon volume regarding some procedures/conditions. This is especially true for pancreatic surgery. Another overview also analyzed the relationship between volume and outcomes for both hospital and surgeon/physician volume [58]. The overview was published in Italian which is why we refer to the English abstract. It found a positive association between surgeon volume and outcomes for unruptured AAA and for various cancer surgeries (colon, bladder, breast, esophagus) which is in line with our results. Additionally, the authors found an association for hip arthroplasty, lower extremity bypass surgery, and stomach cancer which were not analyzed in our review as well as for coronary angioplasty and coronary artery bypass whereas we rated the relationship for CABG and for PCI as unclear. To our knowledge, there has been no overview which analyzes the corresponding topic of whether surgeon volume is associated to outcomes if the results are adjusted for hospital volume and vice versa. This might be an interesting approach for future research.

When performing systematic reviews to explore the volume-outcome relationship many methodological issues must be taken into consideration. A vast majority of the included systematic reviews explicitly states that the definition of cut-off values for the volume groups differed widely among the different primary studies. This problem occurs for all analyzed procedures/conditions. The same amount of performed surgeries can be defined either as low or high volume [59], e.g., depending on the geographical area. This can make findings across studies difficult to compare, and this has to be taken into account in conducting systematic reviews. Moreover, the rationale for specific cut-off values was only explained rarely. In addition, surgeon volume can be defined in several ways. Annual volumes can be pooled over a given time span to calculate an annual mean [5]. Others calculate annual caseloads by taking the number of surgeries by the surgeon during the calendar year [60]. For hospital volume-outcome analyses, it has been shown that conclusions are similar regardless of how hospital volume was defined [61]. For us, there are no obvious reasons why this should differ with respect to surgeon volume. Nevertheless, it should be mentioned that reporting of definitions of volume was inadequate and not explicitly presented within many of the included systematic reviews.

In addition to that, analyzed outcomes were not sufficiently defined in some of the included systematic reviews. Some reviews refrained from specifying which kind of mortality [29, 41, 53] (e.g., postoperative, in-hospital, 30-day, 90-day) or survival [30, 32, 42, 52] (e.g., 5-year overall, 5-year disease-specific) was measured in their included primary studies. Likewise, there was a lack of reporting on definitions of other outcomes (e.g., complications).

Results of different studies should only be pooled quantitatively if the studies use similar interventions, patients, and measures of outcomes so that clinical homogeneity exists [62]. Several systematic reviews refrained from stating that they did not pool different interventions [26, 31, 32, 46, 49, 51, 52]. Additionally, the volume categories differed across primary studies although their results were pooled quantitatively. Some reviews [25, 31, 35, 46, 52] pooled results although *I* ^2^ was bigger than 75% indicating high statistical heterogeneity [63].

Moreover, it should be mentioned that the methodological evaluation of the systematic review about the Norwood procedure might not be completely objective as two authors of this overview (DP and TM) authored the respective review.

We performed an evidence synthesis based on systematic reviews instead of primary studies. This has some implications when interpreting our results. We did not critically appraise the quality of primary studies but relied on the judgements made by review authors. To overcome this, we applied strict inclusion criteria for systematic reviews. We conducted our evidence synthesis based on the procedures/conditions reported within the included systematic reviews. However, results might be more valid if they were reported only on the procedure level as different procedures might be mixed on the condition level. Nevertheless, we think that within our work it is appropriate to summarize results as reported within the included systematic reviews. By doing so, we were able to give an overview of the volume–outcome relationship on many different procedures/conditions. We applied modified vote counting to present results of narrative synthesis. This turned out to be difficult for many reviews due to missing information in included reviews. In addition, recently published primary studies might not have been included in our identified systematic reviews. However, it was our intention to identify possible evidence gaps to present the current state of synthesized evidence and show the potential for updating systematic reviews. Although there is currently little empirical evidence on updating systematic reviews [64], approximately half of the reviews are out of date after 5.5 years, though it must be acknowledged that this estimate stems from systematic reviews of randomized controlled trials and might therefore not necessarily hold true for systematic reviews of observational studies [65]. Based on this assumption, there might be a lack of sound and up-to-date reviews in AAA and in breast cancer as the included most up-to-date reviews for these conditions were published before 2011. We are aware of primary studies that were published after the last published systematic review on AAA [66] and on breast cancer [67, 68]. For all other procedures/conditions, the respective most up-to-date reviews were published in 2011 or later. Nevertheless, we are also aware of primary studies published after the last published review for cystectomy [69, 70] and lung cancer [71]. This might be relevant as the body of evidence for both procedures/conditions is limited based on existing systematic reviews.

We believe that our results will also help to conduct methodologically more sound reviews. Future systematic reviews should consider that cut-off values for the volume groups differ among different primary studies, and this should be considered especially when pooling results. Moreover, different definitions of outcomes among primary studies should be recorded within systematic reviews and considered when pooling results or when making conclusions. Taking into account our assessment of the reviews’ methodological quality, future reviews should especially pay attention to the assessment and documentation of the scientific quality of the primary studies and to the consideration of the scientific quality when formulating conclusions. It means that review authors should explicitly state how scientific quality of included primary studies was assessed, present the results of the assessment for each included study, and consider these results when formulating conclusions.

It has been questioned whether administrative data is as good as clinical data to explore the volume-outcome relationship [72]. Risk adjustment using administrative data has been shown to lead to higher differences in effects between high-volume and low-volume surgeons than using clinical data [73]. Clinical case-mix imbalances related to surgeon volume should be considered and adjusted for in previous studies in addition to administrative risk adjustments as they might be an important confounding variable. Another problem related to data is the multiple uses of the same datasets. Only very few of our reviews considered data quality and the possibility of overlapping data of primary studies.

## Conclusions

When taking into account its limitations, this overview can serve as an informational basis for decision makers (political and institutional leaders) thinking about the importance of surgeon volume regarding quality in health care. Our results seem to support a positive volume-outcome relationship for most procedures/conditions especially in colorectal cancer, bariatric surgery, and breast cancer. However, results are partly based on systematic reviews with methodological weaknesses, e.g., the lack of consideration of the risk of bias in the primary studies. Forthcoming reviews should pay more attention to methodology specific to volume-outcome relationship. Our work can be useful for considerations about minimum volume thresholds of surgeries performed by single surgeons. Nevertheless, the calculation of minimum volume thresholds lies beyond the scope of the review and needs further research.

## Additional files

Additional file 1: PRISMA 2009 Checklist. Completed PRISMA checklist for this work. (DOCX 31 kb)
Additional file 2: Search strategies for medical databases. Search strategies for medical databases used within this systematic review of systematic reviews. (DOCX 17 kb)
Additional file 3: Searched health technology assessment organizations. Health technology assessment organization that were members of INAHTA, HTAi, or EUnetHTA. (DOCX 21 kb)
Additional file 4: Items of AMSTAR. Items used to assess methodological quality of included systematic reviews (includes items of AMSTAR and one additional item). (DOCX 15 kb)
Additional file 5: List of excluded studies. Publications excluded during full-text screening ordered by exclusion criteria. (DOCX 25 kb)
Additional file 6: Study characteristics of included systematic reviews. Study characteristics of included systematic reviews including the analyzed procedure/condition, the inclusion criteria of the systematic reviews, and the number of primary studies included per systematic review. (DOCX 45 kb)
Additional file 7: Results of included systematic reviews. Results of included systematic reviews including pooled results for meta-analyses or vote counting for narrative systematic reviews. (DOCX 63 kb)

## Abbreviations

AAA: Abdominal aortic aneurysm
CABG: Coronary artery bypass graft
CCA: Corrected covered area
CDSR: Cochrane Database of Systematic Reviews
EUnetHTA: European Network for Health Technology Assessment
HR: Hazard ratio
HTAi: Health Technology Assessment international
HVS: High-volume surgeons
INAHTA: The International Network of Agencies for Health Technology Assessment
OR: Odds ratio
PCI: Percutaneous coronary intervention
