# Supplementary material for: Relationship between surgeon volume and outcomes: a systematic review of systematic reviews
Source: Syst Rev. 2016 Nov 29;5:204. doi: 10.1186/s13643-016-0376-4 (PMC5129247; doi:10.1186/s13643-016-0376-4)
Supplement: Additional file 2: — Search strategies for medical databases. Search strategies for medical databases used within this systematic review of systematic reviews. (DOCX 17 kb) [file 13643_2016_376_MOESM2_ESM.docx]

## Additional file 2: Search strategies for medical databases

### Medline (via Pubmed)

((((“Meta-Analysis“ [Publication Type] OR “Meta-Analysis as Topic” [Mesh] OR meta analy* [TIAB] OR metaanaly* [TIAB] OR systematic review* [TIAB] OR systematic literature review* [TIAB] OR systematic overview* [TIAB] OR “Review Literature as Topic” [Mesh] OR pubmed [TIAB] OR medline [TIAB] OR cochrane [TIAB] OR embase [TIAB] OR psychlit [TIAB] OR psyclit [TIAB] OR psychinfo [TIAB] OR psycinfo [TIAB] OR cinahl [TIAB] or science citation index [TIAB] OR cancerlit [TIAB] OR reference list* [TIAB] OR bibliograph* [TIAB] OR hand-search* [TIAB] OR relevant journals [TIAB] OR manual search* [TIAB] OR ((selection criteria [TIAB] OR inclusion criteria [TIAB] OR data extraction [TIAB] AND review [publication type])) NOT ((“Comment” [Publication Type] OR “Letter” [Publication Type] OR “Editorial” [Publication Type]) OR (“animals” [Mesh] NOT (“animals” [Mesh] AND “humans” [Mesh]))))))) AND (((((((((((((((((caseload*) OR workload) OR workload [mesh]) OR "provider volume") OR "procedural volume") OR "surgical volume") OR "volume standard") OR "volume outcome") OR "surgery volume") OR "operator volume") OR "surgeon volume") OR "physician volume") OR "doctor volume") OR "medical practitioner volume") OR "individual volume") OR "personal volume") OR "private volume")

### Embase (via Embase)

('meta analysis'/exp OR 'systematic review'/exp OR (meta NEAR/1analy*):ab,ti OR metaanalys*:ab,ti OR (systematic NEAR/1 (review* OR overview*)):ab,ti OR 'systematic literature Review':ab,ti OR pubmed:ab OR medline:ab OR Cancerlit:ab OR cochrane:ab OR embase:ab OR psychlit:ab OR psyclit:ab OR psychinfo:ab OR psycinfo:ab OR cinahl:ab OR cinhal:ab OR ‘science citation index’:ab OR ‘reference lists’:ab OR bibliograph*:ab OR (hand NEXT/1 search*):ab OR (manual NEXT/1 search*):ab OR ‘relevant journals’:ab OR ((‘data extraction’ OR ‘selection criteria’ OR ‘inclusion criteria’):ab AND review:it) NOT (Letter:it OR editorial:it OR ((‘animal’/exp OR ‘animal experiment’/exp OR ‘animal model’/exp OR ‘nonhuman’/exp) NOT ((‘animal’/exp OR ‘animal experiment’/exp OR ‘animal model’/exp OR ‘nonhuman’/exp) AND ‘human’/exp)))) AND (caseload* OR ‘workload’ OR ‘workload’/exp OR workload OR ‘provider volume’ OR ‘procedural volume’ OR ‘surgical volume’ OR volume NEXT/1 standard* OR volume NEXT/1 outcome OR ‘surgery volume’ OR ‘operator volume’ OR ‘surgeon volume’ OR ‘physician volume’ OR ‘doctor volume’ OR ‘medical practitioner volume’ OR ‘individual volume’ OR ‘personal volume’ OR ‘private volume’)

### Cochrane Database of Systematic Reviews (via Wiley Online Library)

caseload? OR workload OR “provider volume” OR “procedural volume” OR “surgical volume” OR “volume outcome” OR “surgery volume” OR “operator volume” OR “surgeon volume” OR “physician volume” OR “doctor volume” OR “medical practitioner volume” OR “individual volume” OR “personal volume” OR “private volume”
