# Supplementary material for: Relationship between surgeon volume and outcomes: a systematic review of systematic reviews
Source: Syst Rev. 2016 Nov 29;5:204. doi: 10.1186/s13643-016-0376-4 (PMC5129247; doi:10.1186/s13643-016-0376-4)
Supplement: Additional file 3: — Searched health technology assessment organizations. Health technology assessment organization that were members of INAHTA, HTAi, or EUnetHTA. (DOCX 21 kb) [file 13643_2016_376_MOESM3_ESM.docx]

## Additional file 3: Searched health technology assessment organizations

AAZ (Agency for Quality and Accreditation in Health Care)

AETMIS (Agence d’Évaluation des Technologies et des Modes d’Intervention en Santé)

AETS ICS III (Agencia de Evaluación de Tecnologías Sanitarias)

AETSA (Andalusian Agency for Health Technology Assessment)

Age.Na:s (Agenzia Nazionale per I Servizi Sanitari Regionali)

AIFA (Agenzia Italiana Del Farmaco)

AHRQ (US Agency for Healthcare Research and Quality)

AHTA (Adelaide Health Technology Assessment)

AHTAPol (Agency for Health Technology Assessment in Poland)

ARESS (Agenzia Regionale per I Servizi Sanitari)

ARSENÁL (Veneto’s Research Centre for e-Health Innovation)

ASERNIP-S (Australian Safety and Efficacy Register of New Interventional Procedures - Surgical)

ASSR (Regione Emilia Romagna, Agenzia Sanitaria e Sociale Regione Emilia Romagna)

AVALIA-T (Galician Agency for Health Technology Assessment)

BAG (Bundesamt für Gesundheit) / FOPH (Federal Office of Public Health)

BCBS (Blue Cross BlueShield Association)

BS-CA (Blue Shield of California Foundation)

CADTH (Canadian Agency for Drugs and Technologies in Health)

CAHIAQ (Catalan Agency for Health Information, Assessment and Quality) (formerly CAHTA)

CAST (Centre for Applied Health Services Research and Technology Assessment, University of Southern Denmark)

CDE (Center for Drug Evaluation)

CEDIT (Comité d’Évaluation et de Diffusion des Innovations Technologiques)

CEM (Cellule d’expertise médicale)

CENETEC (Centro Nacional de Excelencia Tecnológica en Salud)

CMeRC (Charlotte Maxeke Research Consortium - HTA Unit)

CMTP (Center for Medical Technology Policy)

CNHTA (Committee for New Health Technology Assessment)

CRD (Centre for Reviews and Dissemination)

CVZ (College voor Zorgverzekeringen, Dutch health care insurance board)

DACEHTA (Danish Centre for Health Technology Assessment)

DAHTA@DIMDI (Deutsche Agentur für Health Technology Assessment - Bewertung gesundheitsrelevanter Verfahren - Deutsches Institut für medizinische Dokumentation und Information)

DECIT-CGATS (Secretaria de Ciência, Tecnologia e Insumos Estratégicos, Departamento de Ciência e Tecnologia)

DSI (Danish Institute for Health Services Research)

EMKI (Institute for Healthcare Quality Improvement and Hospital Engineering)

ESKI (National Institute for Strategic Health Research)

ETESA (Department of Quality and Patient Safety of the Ministry Health of Chile)

FEGAS (School of Health Administration)

FIMEA (Finnish Medicines Agency)

FinOHTA (Finnish Office for Health Technology Assessment)

G-BA (Gemeinsamer Bundesausschuss)

GÖG/BIQG (Gesundheit Österreich GmbH)

GR (Gezondheidsraad)

GYEMSZI (National Institute for Quality and Organizational Development in Healthcare and Medicines)

HA (Hospital authority Hong Kong)

HAS (Haute Autorité de Santé)

HIS (Health Care Improvement Scotland)

HIQA (Health Information and Quality Authority)

HITAP (Health Intervention and Technology Assessment Program)

HSAC (Health Services Assessment Collaboration)

HTA-HSR/DHTA (HTA & Health Services Research)

HVB (Hauptverband der Österreichischen Sozialversicherungsträger)

ICER (Institute for Clinical and Economic Review)

ICTAHC (Israel Center for Technology Assessment in Health Care)

IECS (Institute for Clinical Effectiveness and Health Policy)

IER (Institute for Economic Research)

IHE (Institute of Health Economics)

INESSS (Institut national d’excellence en santé et en services)

INFARMED (National Authority of Medicines and Health Products)

IPP (Institut für Public Health und Pflegeforschung, Universität Bremen)

IQWiG (Institut für Qualität und Wirtschaftlichkeit im Gesundheitswesen)

IRF (Institute for Rational Pharmacotherapy)

JAZMP (Agency for Medicinal Products and Medical Devices)

KCE (Belgian Federal Health Care Knowledge Centre)

KDTD (Turkish Evidence-Based Medicine Association)

JAZMP (Agency for Medicinal Products and Medical Devices)

Kaiser Permanente

Kela (The Social Insurance Institution of Finland)

Laziosanità (Agenzia di Sanità Pubblica, Regione Lazio)

LBI (Ludwig Boltzmann Institut for Health Technology Assessment)

MaHTAS (Health Technology Assessment Section, Ministry of Health Malaysia)

MHRA (Medicines and Healthcare Products Regulatory Agency)

MAS (Medical Advisory Secretariat, within the Ontario Ministry of Health and Long-Term Care Health Strategies Division)

MOH CZ (Ministry of Health - Czech Republic)

MOH RS (Ministry of Health - Serbia)

MOH Spain (Ministry of Health - Spain)

MOH Indonesia (Ministry of Health - Republic of Indonesia)

MOH Singapore (Ministry of Health - Singapore)

MSAC (Medical Services Advisory Committee)

MTAA (Medical Technologies Association of Australia)

MTU-SFOPH (Medical Technology Unit - Swiss Federal Office of Public Health)

NBoH (National Board of Health)

NCPE (National Centre for Pharmacoeconomics, St James’s Hospital)

NCPHP (National Centre of Public Health Protection)

NECA (National Evidence-based healthcare Collaboration Agency)

NETSCC, HTA - NIHR (Coordinating Centre for Health Technology Assessment)

Newcastle University

NHG (National Healthcare Group)

NICE (National Institute for Clinical Excellence)

NIPH-RS (National Institute of Public Health of the Republic of Slovenia)

NLM (National Library of Medicine)

NSPH (National School of Public Health)

NHSC (National Horizon Scanning Centre)

NHS QIS (Quality Improvement Scotland)

NOKC (Norwegian Knowledge Centre for the Health Services)

OSTEBA (Basque Office for Health Technology Assessment)

PATH (Programs for Assessment of Technology in Health Research Institute)

PBAC (Australian Government, Department of Health & Ageing)

PenTAG (Peninsula Technology Assessment Group)

PHARMAC (Pharmaceutical Management Agency of New Zealand)

QPACT (Queensland Policy and Advisory Committee for New Technology)

Regione Veneto (Regione Veneto, Direzione Piani e Programmi Socio Sanitari)

Reglom-DGSAN (Regione Lombardia Direzione Generale Sanita)

RIZIV (Rijksinstituut voor ziekte - en invaliditeitsverzekering)

santésuisse (Branchenverband der schweizerischen Krankenversicherer)

SBU (Swedish Council on Technology Assessment in Health Care)

ScHARR (Technology Assessment Group, University of Sheffield)

SIDC (State Institute for Drug Control)

SingHealth (Singapore Health Service)

SLOVATHA (Slovak Agency for Health Technology Assessment)

SNHTA (Swiss Network for HTA)

SNSPMS (National School of Public Health, Management and Professional Development)

SPC on Standardization and HTA

SSD/MSOC (Ministry for Social Policy, Strategy and Sustainability Division)

Sundhed.dk (Centre for Public Health, Central Denmark Region, department HTA & Health Services Research)

TLV (Dental and Pharmaceutical Benefits Agency)

UCEETS (The National Coordination Unit of Health Technology Assessment and Implementation)

UETS (Unidad de Evaluación de Tecnologías Sanitarias)

UFI-SALUD (Unidad de Financiamiento Internacional de Salud)

UMIT (Private Universität für Gesundheitswissenschaften, Medizinische Informatik und Technik)

University Hospital A Gemelli

UTA (University of Tartu, Department of Public Health)

UVT (HTA Unit in A Gemelli Teaching Hospital)

VASPVT (State Health Care Accreditation Agency under the Ministry of Health of the Republic of Lithuania)

VATAP (VA Technology Assessment Program)

VEC (Centre of Health Economics)

ZonMw (The Medical and Health Research Council of the Netherlands)
