# Supplementary material for: Relationship between surgeon volume and outcomes: a systematic review of systematic reviews
Source: Syst Rev. 2016 Nov 29;5:204. doi: 10.1186/s13643-016-0376-4 (PMC5129247; doi:10.1186/s13643-016-0376-4)
Supplement: Additional file 4: — Items of AMSTAR. Items used to assess methodological quality of included systematic reviews (includes items of AMSTAR and one additional item). (DOCX 15 kb) [file 13643_2016_376_MOESM4_ESM.docx]

## Additional file 4: Items of AMSTAR

1. Was an ‘a priori’ design provided?

2. Was there duplicate study selection and data extraction?

3. Was a comprehensive literature search performed?

4. Was the status of publication (i. e., grey literature) used / not used as an inclusion criterion?

5. Was a list of studies (included and excluded) provided?

6. Were the characteristics of the included studies provided?

7. Was the scientific quality of the included studies assessed and documented?

8. Was the scientific quality of the included studies used appropriately in formulating conclusions?

9. Were the methods used to combine the findings of studies appropriate?

10. Was the likelihood of publication bias assessed?

11. Was the conflict of interest stated?

12. Was dealing with multiple comparisons in primary studies stated?
