# Supplementary material for: Relationship between surgeon volume and outcomes: a systematic review of systematic reviews
Source: Syst Rev. 2016 Nov 29;5:204. doi: 10.1186/s13643-016-0376-4 (PMC5129247; doi:10.1186/s13643-016-0376-4)
Supplement: Additional file 5: — List of excluded studies. Publications excluded during full-text screening ordered by exclusion criteria. (DOCX 25 kb) [file 13643_2016_376_MOESM5_ESM.docx]

**Additional file 5: List of excluded studies**

Exclusion criteria 1: No systematic review on the basis of primary studies

Increased hospital and physician volumes associated with increased survival. Evidence-Based Healthcare and Public Health 2004;8:255-256.

Birkmeyer JD, Dimick JB. Potential benefits of the new Leapfrog standards: Effect of process and outcomes measures. Surgery 2004;135:569-575.

Davoli M, Amato L, Minozzi S, Bargagli AM, Vecchi S, Perucci CA. Volume and health outcomes: an overview of systematic reviews. Epidemiologia e prevenzione 2005;29:3-63.

Flynn K. Regionalization of surgical services (Structured abstract). Health Technology Assessment Database [Internet]. 2009; (3). Available from: <http://onlinelibrary.wiley.com/o/cochrane/clhta/articles/HTA-32010001506/frame.html>.

Karthikesalingam A, Hinchliffe RJ, Loftus IM, Thompson MM, Holt PJ. Volume-outcome relationships in vascular surgery: The current status. Journal of Endovascular Therapy 2010;17:356-365.

Moore Sarah E, Raval Manoj J, Phang Terry P, Bressler B, Brown Carl J. Surgery for ulcerative colitis: Outcomes in low volume hospitals versus high volume hospitals. Cochrane Database of Systematic Reviews [Internet]. 2012; (10). Available from: <http://onlinelibrary.wiley.com/doi/10.1002/14651858.CD010175/abstract>.

Ohmann C, Blum K, De Cruppe W, Geraedts M. Hospital volume and outcome: Is one parameter sufficient for assessment? Chirurgische Gastroenterologie Interdisziplinär 2008;24:281-286.

Paterson-Brown S. Surgical volume and clinical outcome. British Journal of Surgery 2007;94:523- 524.

Taylor I. Surgical issues in rectal cancer. Annals of Oncology 2008;19:viii41-viii42.

Tol JAMG, Van Gulik TM, Busch ORC, Gouma DJ. Centralization of highly complex low-volume procedures in upper gastrointestinal surgery. A summary of systematic reviews and meta- analyses. Digestive Surgery 2012;29:374-383.

Vrijens F, De Gauquier K, Camberlin C. The volume of surgical interventions and its impact on the outcome: feasibility study based on Belgian data. Brussels: Belgian Health Care Knowledge Centre (KCE), 2009.

Williams OD. Quality of care versus provider volume: does one lead to the other? Medical care 2003;41:1127-1128.

Wouters MWJM, Jansen-Landheer MLEA, Van De Velde CJH. The quality of cancer care initiative in the Netherlands. European Journal of Surgical Oncology 2010;36:S3-S13.

Exclusion criteria 2: No volume-outcomes relationship in surgery

Ahmed S, Luks FI, O'Brien BM, Muratore CS, Carr SR. Influence of experience, case load, and stage distribution on outcome of endoscopic laser surgery for TTTS: A review. Prenatal Diagnosis 2010;30:314-319.

Bilimoria KY, Phillips JD, Rock CE, Hayman A, Prystowsky JB, Bentrem DJ. Effect of surgeon training, specialization, and experience on outcomes for cancer surgery: A systematic review of the literature. Annals of Surgical Oncology 2009;16:1799-1808.

Gandjour A, Lauterbach KW. The relationship between provider volume and mortality rate: Volume data of German centres of excellence. Zeitschrift für Kardiologie 2001;90:613-620.

Kanhere MH, Kanhere HA, Cameron A, Maddern GJ. Does patient volume affect clinical outcomes in adult intensive care units? Intensive Care Medicine 2012;38:741-751.

Obladen M. Minimum patient volume in the care for very low birthweight infants: A review of the literature. Zeitschrift fur Geburtshilfe und Neonatologie 2007;211:110-117.

Post PN, Wittenberg J, Burgers JS. Do specialized centers and specialists produce better outcomes for patients with chronic diseases than primary care generalists? A systematic review. International Journal for Quality in Health Care 2009;21:387-396.

Exclusion criteria 3: No definition of patient group or indication

Chowdhury MM, Dagash H, Pierro A. A systematic review of the impact of volume of surgery and specialization on patient outcome. British Journal of Surgery 2007;94:145-161.

Exclusion criteria 4: Only investigation of hospital volume

Relationship between volume and quality of health care: a review of the literature (Structured abstract). Database of Abstracts of Reviews of Effects [Internet]. 1995; (3):[34 p.]. Available from: <http://onlinelibrary.wiley.com/o/cochrane/cldare/articles/DARE-> 11995002733/frame.html.

Awopetu AI, Moxey P, Hinchliffe RJ, Jones KG, Thompson MM, Holt PJE. Systematic review and meta-analysis of the relationship between hospital volume and outcome for lower limb arterial surgery. British Journal of Surgery 2010;97:797-803.

Henebiens M, van den Broek TAA, Vahl AC, Koelemay MJW. Relation between Hospital Volume and Outcome of Elective Surgery for Abdominal Aortic Aneurysm: A Systematic Review. European Journal of Vascular and Endovascular Surgery 2007;33:285-292.

Holt PJE, Poloniecki JD, Gerrard D, Loftus IM, Thompson MM. Meta-analysis and systematic review of the relationship between volume and outcome in abdominal aortic aneurysm surgery. British Journal of Surgery 2007;94:395-403.

Holt PJE, Poloniecki JD, Loftus IM, Thompson MM. Meta-Analysis and Systematic Review of the Relationship between Hospital Volume and Outcome Following Carotid Endarterectomy. European Journal of Vascular and Endovascular Surgery 2007;33:645-651.

La Torre M, Nigri G, Ferrari L, Cosenza G, Ravaioli M, Ramacciato G. Hospital volume, margin status, and long-term survival after pancreaticoduodenectomy for pancreatic adenocarcinoma. American Surgeon 2012;78:225-229.

Lüngen M, Rupprecht CJ, Plamper E, Lauterbach KW. Centralisation of breast cancer management by giving minimum work-load. Empirical effects in the region of North-Rhine, Germany. Zeitschrift für ärztliche Fortbildung und Qualitätssicherung 2004;98:385-389.

Markar SR, Karthikesalingam A, Thrumurthy S, Low DE. Volume-outcome relationship in surgery for esophageal malignancy: systematic review and meta-analysis 2000-2011. Journal of gastrointestinal surgery : official journal of the Society for Surgery of the Alimentary Tract 2012;16:1055-1063.

NHS Centre for Reviews and Dissemination. Relationship between volume and quality of health care: a review of the literature. York: 1995.

Pettit SJ, Jhund PS, Hawkins NM, Gardner RS, Haj-Yahia S, McMurray JJV, et al. How small is too small? A systematic review of center volume and outcome after cardiac transplantation. Circulation: Cardiovascular Quality and Outcomes 2012;5:783-790.

Post PN, Kuijpers M, Ebels T, Zijlstra F. The relation between volume and outcome of coronary interventions: a systematic review and meta-analysis. European Heart Journal 2010;31:1985- 1992.

Richardson AJ, Pang TCY, Johnston E, Hollands MJ, Lam VWT, Pleass HCC. The Volume Effect in Liver Surgery - A Systematic Review and Meta-analysis. Journal of Gastrointestinal Surgery 2013;17:1984-1996.

Sowden AJ, Deeks JJ, Sheldon TA. Volume and outcome in coronary artery bypass graft surgery: true association or artefact? BMJ 1995;311:151-155.

Tjarda Van Heek N, Kuhlmann KFD, Scholten RJ, De Castro SMM, Busch ORC, Van Gulik TM, et al. Hospital volume and mortality after pancreatic resection: A systematic review and an evaluation of intervention in The Netherlands. Annals of Surgery 2005;242:781-790.

Troeng T. Volume versus outcome when treating abdominal aortic aneurysm electively - is there evidence to centralise? Scandinavian Journal of Surgery 2008;97:154-159; discussion 159- 160.

Vernooij F, Heintz P, Witteveen E, Graaf Y. The outcomes of ovarian cancer treatment are better when provided by gynecologic oncologists and in specialized hospitals: a systematic review (Structured abstract). Gynecologic Oncology [Internet]. 2007; (3):[801-812 pp.]. Available from: <http://onlinelibrary.wiley.com/o/cochrane/cldare/articles/DARE-> 12007001883/frame.html.

Exclusion criteria 5: Other language than English or German

Agence d’évaluation des technologies et des modes d’intervention en santé (AETMIS). Traitement chirurgical du cancer de l’œsophage: effet du volume d’interventions sur les résultats cliniques. ETMIS 2011;7:1-45.

Amato L, Colais P, Davoli M, Ferroni E, Fusco D, Minozzi S, et al. [Volume and health outcomes: evidence from Systematic Reviews and from evaluation of Italian hospital data]. Epidemiologia & Prevenzione 2013;37:1-100.

Boughrassa F, Framarin A. Surgical treatment of esophageal cancer: effect of operative volume on clinical outcomes (Structured abstract). Health Technology Assessment Database [Internet]. 2011; (3). Available from: <http://onlinelibrary.wiley.com/o/cochrane/clhta/articles/HTA-> 32011001606/frame.html.

Slim K, Flamein R, Chipponi J. Relationship between surgeon's volume and outcomes: Myth or reality? Annales de Chirurgie 2002;127:502-511.

Teisberg P, Hansen FH, Hotvedt R, Ingebrigtsen T, Kvalvik AG, Lund E, et al. Hospital volume and quality of health outcome (Structured abstract). Health Technology Assessment Database [Internet]. 2001; (3). Available from: http://onlinelibrary.wiley.com/o/cochrane/clhta/articles/HTA-32001000173/frame.html.

Van Heek NT, Kuhlmann KFD, Scholten RJPM, De Castro SMM, Busch ORC, Van Gulik TM, et al. Centralisation of pancreatic resection: A systematic review and evaluation in the Netherlands. Nederlands Tijdschrift voor Geneeskunde 2006;150:791-798.

**Exclusion criteria 6: Results not separated for surgeon / hospital volume**

Killeen SD, Andrews EJ, Redmond HP, Fulton GJ. Provider volume and outcomes for abdominal aortic aneurysm repair, carotid endarterectomy, and lower extremity revascularization procedures. Journal of Vascular Surgery 2007;45:615-626.

Killeen SD, O'Sullivan MJ, Coffey JC, Kirwan WO, Redmond HP. Provider volume and outcomes for oncological procedures. British Journal of Surgery 2005;92:389-402.

Lüngen M, Lauterbach KW. The effectiveness of structural quality in quality assurance. A review. Zeitschrift für ärztliche Fortbildung und Qualitätssicherung 2002;96:101-114.

Mayer EK, Purkayastha S, Athanasiou T, Darzi A, Vale JA. Assessing the quality of the volume- outcome relationship in uro-oncology. BJU International 2009;103:341-349.

Schrader P, Rath T. Mimimum requirements in total knee replacement. Evidence report and model calculation of the healthcare situation. Orthopäde 2005;34:198-209.

Schrader P, Rath T. Volume-outcome-relationship in total hip replacement--literature review and model calculation of the health care situation. Zeitschrift für Orthopädie und Unfallchirurgie 2007;145:281-290.

Exclusion criteria 7: No systematic evaluation of the methodological quality of included primary studies

Barocas DA, Mitchell R, Chang SS, Cookson MS. Impact of surgeon and hospital volume on outcomes of radical prostatectomy. Urologic Oncology: Seminars and Original Investigations 2010;28:243-250.

Bollschweiler E, Metzger R, Vallbohmer D, Holscher AH. Minimum case loads in visceral surgery - What is crucial: The surgical center or the single surgeon? Chirurgische Gastroenterologie Interdisziplinär 2008;24:274-279.

Critchley RJ, Baker PN, Deehan DJ. Does surgical volume affect outcome after primary and revision knee arthroplasty? A systematic review of the literature. Knee 2012;19:513-518.

du Bois A, Rochon J, Pfisterer J, Hoskins WJ. Variations in institutional infrastructure, physician specialization and experience, and outcome in ovarian cancer: a systematic review. Gynecologic Oncology 2009;112:422-436.

Hebert-Croteau N, Roberge D, Brisson J. Provider's volume and quality of breast cancer detection and treatment. Breast Cancer Research and Treatment 2007;105:117-132.

Joudi FN, Konety BR. The volume/outcome relationship in urologic cancer surgery. Supportive Cancer Therapy 2004;2:42-46.

Joudi FN, Konety BR. The impact of provider volume on outcomes from urological cancer therapy. Journal of Urology 2005;174:432-438.

Kalant N, Shrier I. Volume and outcome of coronary artery bypass graft surgery: Are more and less the same? Canadian Journal of Cardiology 2004;20:81-86.

Lauder CIW, Marlow NE, Maddern GJ, Barraclough B, Collier NA, Dickinson IC, et al. Systematic review of the impact of volume of oesophagectomy on patient outcome. ANZ Journal of Surgery 2010;80:317-323.

Mahar AL, McLeod RS, Kiss A, Paszat L, Coburn NG. A systematic review of the effect of institution and surgeon factors on surgical outcomes for gastric cancer (Structured abstract). Journal of the American College of Surgeons [Internet]. 2012; (5):[860-868.e812 pp.]. Available from: http://onlinelibrary.wiley.com/o/cochrane/cldare/articles/DARE-12012022287/frame.html.

Markar SR, Penna M, Karthikesalingam A, Hashemi M. The impact of hospital and surgeon volume on clinical outcome following bariatric surgery. Obesity Surgery 2012;22:1126-1134.

Marlow NE, Barraclough B, Collier NA, Dickinson IC, Fawcett J, Graham JC, et al. Effect of hospital and surgeon volume on patient outcomes following treatment of abdominal aortic aneurysms: A systematic review. European Journal of Vascular and Endovascular Surgery 2010;40:572- 579+e557+e563.

Marlow NE, Barraclough B, Collier NA, Dickinson IC, Fawcett J, Graham JC, et al. Centralization and the relationship between volume and outcome in knee arthroplasty procedures. ANZ Journal of Surgery 2010;80:234-241.

Meyer HJ. The influence of case load and the extent of resection on the quality of treatment outcome in gastric cancer. European Journal of Surgical Oncology 2005;31:595-604.

Nugent E, Neary P. Rectal cancer surgery: Volume-outcome analysis. International Journal of Colorectal Disease 2010;25:1389-1396.

Nuttall M, Van Der Meulen J, Phillips N, Sharpin C, Gillatt D, McIntosh G, et al. A systematic review and critique of the literature relating hospital or surgeon volume to health outcomes for 3 urological cancer procedures. Journal of Urology 2004;172:2145-2152.

Shackley P, Slack R, Booth A, Michaels J. Is there a positive volume-outcome relationship in peripheral vascular surgery? Results of a systematic review. European Journal of Vascular and Endovascular Surgery 2000;20:326-335.

Shervin N, Rubash HE, Katz JN. Orthopaedic procedure volume and patient outcomes: a systematic literature review. Clinical orthopaedics and related research 2007;457:35-41.

Siess M, Siewert JR. Patient safety in view of volume standards, specialization and regionalization. Deutsche Medizinische Wochenschrift 2005;130:503-507.

Weitz J, Koch M, Friess H, Buchler MW. Impact of volume and specialization for cancer surgery. Digestive Surgery 2004;21:253-261.

Wilson A, Marlow NE, Maddern GJ, Barraclough B, Collier NA, Dickinson IC, et al. Radical prostatectomy: A systematic review of the impact of hospital and surgeon volume on patient outcome. ANZ Journal of Surgery 2010;80:24-29
