# Supplementary material for: Relationship between surgeon volume and outcomes: a systematic review of systematic reviews
Source: Syst Rev. 2016 Nov 29;5:204. doi: 10.1186/s13643-016-0376-4 (PMC5129247; doi:10.1186/s13643-016-0376-4)
Supplement: Additional file 6: — Study characteristics of included systematic reviews. Study characteristics of included systematic reviews including the analyzed procedure/condition, the inclusion criteria of the systematic reviews, and the number of primary studies included per systematic review. (DOCX 45 kb) [file 13643_2016_376_MOESM6_ESM.docx]

**Additional file 6: Study characteristics of included systematic reviews**

| **Study** | **Condition / procedure** | **Inclusion criteria** | **Number of studies**  **(relevant / total)** |
| --- | --- | --- | --- |
| Archampong et al. 2012 [[24](#_ENREF_24)] | Colorectal,  colon and rectal cancer | - studies published since 1990 | 22/54 |
| Archampong et al. 2010 [[25](#_ENREF_25)] | Rectal cancer | - studies published since 1990 | 11/11 |
| Van Gijn et al. 2010 [[48](#_ENREF_48)] | Colon and colorectal cancer | - subject of the study is the surgical treatment of colon cancer, rectal cancer or both  - hospital and / or surgeon volume is an independent variable  - outcome parameter is postoperative mortality and / or survival  - the study does not describe a single hospital or surgeon  - the study uses primary data (e. g. editorials, systematic reviews are excluded)  - published after 1988  - multivariate analyses had to be corrected for at least age and gender  - volume had to be defined as a distinct number or cut-off value; studies that defined volume as ‘specialization’ were excluded | 7/23 |
| Salz et al. 2008 [[43](#_ENREF_43)] | Rectal cancer | - studies include results for rectal cancer  - studies report original data for which bivariate or multivariate results were reported  - studies reporting results without showing effect sizes were also included  - rectal cancer had to be distinguished from other patient groups | 11/22 |
| Iversen et al. 2007 (short-term) [[35](#_ENREF_35)] | Colorectal cancer  (short-term) | - studies with ≥ 500 patients  - studies published since 1992 | 15/35 |
| Iversen et al. 2007 (long-term) [[36](#_ENREF_36)] | Colorectal cancer  (long-term) | - studies with ≥ 500 patients  - studies published since 1992 | 11/34 |
| Zevin et al. 2012 [[54](#_ENREF_54)] | Bariatric surgery | - studies had to report on the effects of annual hospital volumes and / or annual surgeon volumes, and on patient outcomes (mortality, morbidity, complications, rates of readmission, and lengths of stay)  - only original articles were included in this review, no review articles or opinion pieces | 13/24 |
| Padwal et al. 2011 [[41](#_ENREF_41)] | Bariatric surgery | - RCTs examining efficacy / safety of a common contemporary bariatric surgery (i. e., adjustable gastric banding, Roux-en-Y gastric bypass, sleeve gastrectomy) versus another common contemporary surgical comparator or a non-surgical treatment were prioritized for inclusion  - adult or adolescent (11 to 17 years) populations meeting guideline-concordant eligibility for surgery (Class III obesity or medically complicated Class II obesity) and reporting relevant outcomes were included in the clinical review  - similarly cost-utility or cost-minimization studies and studies examining bariatric surgery volume-outcome relationships were included | 8/14 |
| Klarenbach et al. 2010 [[37](#_ENREF_37)] | Bariatric surgery | - comparative studies  - obese adults (16 years and older) | 7/17 |
| Young et al. 2007 [[53](#_ENREF_53)] | Abdominal aortic aneurysm | - investigation of surgeon volume and outcome, not only hospital volume  - only abdominal aortic aneurysm, not ruptured aneurysms, thoracic or thoracoabdominal aortic aneurysm repairs | 14/14 |
| Wilt et al. 2006 [[50](#_ENREF_50)] | Abdominal aortic aneurysm | - the report had to be an original analysis of data representing repair of unruptured AAA in the endovascular era  - published after 1990  - the report had to represent practices in the United States  - the sample had to represent variation between hospitals or surgeons in a community or larger geographic area, thereby excluding single site cases series  - the report had to present sample statistics (e. g., percentages, odds ratios) representing the relationship between a measure of hospital or physician volume and any good or bad outcome associated with AAA repair  - the analysis had to attempt to make adjustments for known risk factors in an effort to reduce bias | 4/8 |
| Brusselaers et al. 2014 [[26](#_ENREF_26)] | Esophagectomy for cancer | - published after January the first 1990  - original data on survival of patients who underwent esophagectomy for malignancy  - abstracts or other conference proceedings, case reports, case series, intervention studies, and review articles were excluded  - both prospective and retrospective studies were eligible  - articles describing esophagectomy for non-malignant reasons were excluded, as were studies reporting a subgroup of esophagectomy patients only  - if studies also reported survival after gastric cancer surgery, survival for esophageal cancer had to be reported separately  - language restriction was applied only in the end stage of the search, to enable assessment of language selection bias; a priori eligible were English, French, Dutch, German, Spanish, Swedish and Chinese  - studies were eligible only if HRs comparing survival after esophagectomy by hospital or surgeon volume groups, or by hospital type were reported  - minimum reported follow-up time was three months | 4/16 |
| Wouters et al. 2012 [[52](#_ENREF_52)] | Esophageal cancer | - published after January the first 1995  - English language  - the study used primary data (i. e, letters, editorials, and reviews were excluded)  - the subject of the study was the surgical treatment of esophageal cancer  - the study did not describe the results of a single hospital or surgeon  - comparisons between providers (hospitals or surgeons)  - definition for procedural volume as a distinct number or cut-off value (i. e, studies that defined volume as ‘‘specialization’’ were excluded).  - postoperative morbidity, mortality, survival, or quality of life among outcome parameters | 12/43 |
| Trinh et al. 2013 [[47](#_ENREF_47)] | Radical prostatectomy | - hospital and / or surgeon volume is reported as a predictor variable  - a measurable endpoint is clearly defined (such as mortality, perioperative complications or long-term complications)  - multiple hospitals or surgeons are described | 33/45 |
| Wilt et al. 2008 [[51](#_ENREF_51)] | Radical prostatectomy | - evaluation of the associative hypothesis between provider characteristics and patient outcomes  - control group  - written in English  - information regarding provider characteristics  - published after 1980 | 10/17 |
| Lau et al. 2012 [[38](#_ENREF_38)] | Total knee arthroplasty | - study population had to include patients undergoing primary total knee arthroplasty | 11/11 |
| Stengel et al. 2004 [[45](#_ENREF_45)] | Total knee arthroplasty | - analysis of the relationship of hospital and / or surgeon volume and outcomes of primary or revision total knee arthroplasty  - results had to be distinguishable if not only total knee arthroplasty was analyzed  - clinical or patient centered outcome (mortality or morbidity)  - size of the sample had to be mentioned  - calculation of event rates had to be feasible  - in accordance with the guidelines of the American Medical Association | 4/13 |
| Gooiker et al. 2010 [[30](#_ENREF_30)] | Breast cancer surgery | - study with more than two surgeons  - study begin after 1988  - adjustments for age and sex  - only one study (the one with highest quality) per database included | 7/12 |
| **Sepehripour et al. 2013 [**[**44**](#_ENREF_44)**]** | **Off-pump coronary artery bypass surgery** | **NR** | **3/6** |
| Goossens-Laan et al. 2011 [[32](#_ENREF_32)] | Radical cystectomy for bladder cancer | - study with more than two surgeons  - adjusted for age and sex | 3/10 |
| Eskander et al. 2014 [[28](#_ENREF_28)] | Head and neck cancer | - adult patients with nonendocrine non-skin head and neck cancers treated with either ablative surgery, reconstructive surgery, radiation, or chemoradiation  - physician or hospital volume and an outcome measure (in-hospital mortality, short-term survival, long-term survival, recurrence-free survival, readmission to the hospital, length of stay in the hospital or hospital care costs) must have been described  - review articles, commentaries, and case reports were excluded | 9/17 |
| Van Meyenfeldt et al. 2012 [[49](#_ENREF_49)] | Lung cancer | - written in English  - primary data  - subject: surgical treatment of lung cancer  - comparisons between providers (hospitals or surgeons)  - no single-hospital nor single-surgeon studies  - postoperative mortality or survival as outcome parameters  - distinct cut-off value for procedural volume or clearly defined specialty  - published after January the first 1990 | 2/19 |
| Pieper et al. 2014 [[42](#_ENREF_42)] | Norwood procedure | - the study had a comparative design  - patient outcomes (e. g. mortality, morbidity) were studied  - volume (if applicable) was defined as a distinct number (e. g. continuous variable) or a cut-off value, or specialized hospitals / units were analyzed  - the study did not describe a single hospital or surgeon | 4/10 |
| Gooiker et al. 2011 [[31](#_ENREF_31)] | Pancreatic surgery | - study with more than one surgeon  - study begin after 1988  - adjustment for age, sex and co-morbidity | 3/14 |
| **Strom et al. 2014 [**[**46**](#_ENREF_46)**]** | **Percutaneous coronary intervention** | **- studies which examined patients undergoing PCI (with or without stent placement)**  **- studies which reported the effects of operator-specific volume on patient mortality or morbidity**  **- studies which evaluated annualized volumes as opposed to career volumes**  **- published after January 1977** | **21/23** |
| Caputo et al. 2014 [[27](#_ENREF_27)] | Trauma | - consist of original research addressing the topic of institutional or per surgeon volume on mortality  - include data from Level I trauma centres  - English-language publications addressing American trauma centres  - published after January the first 1976  - available abstracts  - study containing a general trauma population  - exclusion of studies that only considered demographic-specific populations, such as geriatric or paediatric patients, rather than injury characteristics, and studies examining exclusively burns | 4/19 |
| Gruen et al. 2009 [[3](#_ENREF_3)] | Gastrointestinal cancer | - no language constraints | 41*/137* |
| Miyata et al. 2007 [[40](#_ENREF_40)] | Several | - papers written in either English or Japanese were reviewed  - only studies on Japanese populations living in Japan were included  - instances of multiple publications from the same database were excluded, with only the most complete publication selected | 2/13 |
| Gandjour et al. 2003 [[29](#_ENREF_29)] | Several | - articles published in English, Dutch, French, German or Italian  - study with more than two hospitals  - Veteran Health Administration hospitals were excluded  - case-mix adjustments  - study begin after 1989 | 25/76 |
| Halm et al. 2002 [[33](#_ENREF_33)] | Several | - patients primarily treated since 1980  - in English  - studies from single institutions, voluntary registries or other convenience samples were excluded  - articles on trauma, new-born intensive care and organ transplantation were excluded | 45*/135* |
| Hillner et al. 2000 [[34](#_ENREF_34)] | Cancer | - studies dealing with screening or early detection were excluded  - stratified or adjusted for clinical stage | NR |
| McAteer et al. 2013 [[39](#_ENREF_39)] | Several in pediatric surgery | - studies that evaluated only patient characteristics at presentation rather than outcomes of care were excluded  - published since 1980 in English  - hospital or surgeon experience as a predictor variable and any clinical outcome as a response variable | 11/63 |

NR – Not reported

* number of comparisons
