# Supplementary material for: Relationship between surgeon volume and outcomes: a systematic review of systematic reviews
Source: Syst Rev. 2016 Nov 29;5:204. doi: 10.1186/s13643-016-0376-4 (PMC5129247; doi:10.1186/s13643-016-0376-4)
Supplement: Additional file 7: — Results of included systematic reviews. Results of included systematic reviews including pooled results for meta-analyses or vote counting for narrative systematic reviews. (DOCX 63 kb) [file 13643_2016_376_MOESM7_ESM.docx]

**Additional file 7: Results of included systematic reviews**

| **Study** | **Case-mix (adjustment in each study)** | **Results (*meta-analyses* [number of studies/ comparisons for each outcome in brackets before effect measure; pooled effect sizes derived by random-effects-model for HVS unless otherwise specified] or *vote counting* [HVS perform better/** **median effect size/** **statistically significant comparisons/** **total number of comparisons])** | |
| --- | --- | --- | --- |
| **Colorectal Cancer** | | | |
| Archampong et al. 2012 [[24](#_ENREF_24)] | Sex for 5-year survival  Sex for five 5-year disease specific survival  Sex for mortality  Age, sex, and unity for analysis of error for anastomotic leak rate  Age and sex for permanent stoma | Overall 5-year survival 4/NR/2/4  (4) HR 1.14 (1.08 – 1.20), I^2^=26%  *Subtotals*  Colorectal Cancer (2) HR 1.15 (1.09 – 1.22), I^2^=0.0%  Colon Cancer (1) HR 1.19 (1.08 – 1.32)  Rectal Cancer (1) HR 1.01 (0.88 – 1.16)  5-year disease specific survival 2/NR/0/2  (2) HR 1.06 (0.87 – 1.30), I^2^=0.0%  *Subtotals*  Colon Cancer (1) HR 1.01 (0.78 – 1.32)  Rectal Cancer (1) HR 1.14 (0.84 – 1.54)  Inpatient and 30-day mortality 5/NR/3/6  (6) OR 0.77 (0.66 – 0.91), I^2^=56%  *Subtotals*  Colon Cancer (4) OR 0.75 (0.62 – 0.92), I^2^=71%  Rectal Cancer (2) OR 0.86 (0.62 – 1.19), I^2^=0.0%  Anastomotic leak rate 2/NR/0/2  (2) OR 0.64 (0.40 – 1.02), I^2^=0.0%  *Subtotals*  Colon Cancer (1) OR 0.66 (0.36 – 1.21)  Rectal Cancer (1) OR 0.61 (0.29 – 1.28)  Permanent stoma rate 2/NR/2/2  Rectal Cancer (2) OR 0.75 (0.64 – 0.88), I^2^=0.0%  Abdominoperineal excision of the rectum  Rectal Cancer (1) OR 0.20 (0.10 – 0.40) | |
| Archampong  et al. 2010 [[25](#_ENREF_25)] | Age and gender for mortality  Age and gender for permanent stoma rate | Overall survival 2/NR/1/2  (2) HR 1.33 (1.16 – 1.54), I^2^=18%  30-day or postoperative mortality 2/NR/0/2  (2) OR 0.79 (0.59 – 1.06), I^2^=0%  Anastomotic leak rate 3/NR/1/3  (3) OR 0.67 (0.38 – 1.17), I^2^=43%  Local recurrence rate  (1) HR 0.42 (0.18 – 0.83)  Permanent stoma rate 2/NR/2/2  (2) OR 0.75 (0.64 – 0.88), I^2^=0  Abdominoperineal excision rate (including rectosigmoid cancer) 6/NR/4/6  (6) OR 0.58 (0.45 – 0.76), I^2^=67%  Abdominoperineal excision rate (excluding rectosigmoid cancer) 3/NR/2/3  (3) OR 0.51 (0.28 – 0.93), I^2^=85% | |
| Van Gijn  et al. 2010 [[48](#_ENREF_48)] | Age and gender for survival  Age and gender for mortality | Long-term survival 5/NR/2/5  Colon cancer (1) HR 1.52 (1.23 – 1.88)  Colorectal cancer (4) HR 1.15 (1.09 – 1.22), I^2^=0.0%  Postoperative mortality 3/NR/3/3  Colon cancer (1) OR 0.82 (0.68 – 0.99)  Colorectal cancer (2) OR 0.67 (0.53 – 0.84), I^2^=45.6% | |
| Salz  et al. 2008 [[43](#_ENREF_43)] | NR | Overall survival 1/NR/1/2  Cancer-specific survival 2/NR/2(?)/5  2-year mortality 1/NR/1(?)/1  Thirty day postoperative mortality (30-day & in-hospital) 2/NR/2(?)/6  Local recurrence 3/NR/3/4  Complications 1/NR/1(?)/3  Abdominoperineal resections / colostomy rate 4/NR/4(?)/6  Reoperation 0(?)/NR/0/1  Adjuvant radiation rate 0(?)/NR/0/1  Distant metastasis 0/NR/0/1 | |
| Iversen et al. 2007 (short-term) [[35](#_ENREF_35)] | NR | Postoperative mortality  Colon cancer (3) OR 0.50 (0.39 – 0.64), I^2^=85.4%; 3/NR/3/3  Colorectal cancer (4) OR 0.82 (0.54 – 1.24), I^2^=68.0%; 3/NR/1/4  Rectal cancer (4) OR 0.72 (0.44 – 1.17), I^2^=34.3%; 2/NR/1/4  Anastomotic leak  Rectal cancer NR/NR/0/2 | |
| Iversen et al. 2007 (long-term) [[36](#_ENREF_36)] | NR | Overall survival  Colon cancer (1) OR 1.25 (1.16 – 1.34)  Colorectal cancer (2) OR 1.37 (0.94 – 1.99), I^2^=58.2%; 2/NR/1/2  Rectal cancer (4) OR 1.16 (0.88 – 1.54), I^2^=70.0%; 2/NR/1/4  Permanent stoma  Rectal cancer (2) OR 0.75 (0.62 – 0.90), I^2^=10.6%; 2/NR/2/2 | |
| **Bariatric surgery** | | | |
| Zevin  et al. 2012 [[54](#_ENREF_54)] | Age and sex for complications  Age and sex for length of stay | 90-day mortality  Open bariatric procedures including revisions 1/NR/1/1  30-day in-hospital mortality 7/NR/5/7  RYGBP (open) 3/NR/2/3  RYGBP (open & laparoscopic) 4/NR/3/4  Complications within 90 days  RYGBP (open & laparoscopic) 1/NR/1/1  30-day in-hospital complications 3/NR/3/3  RYGBP (open & laparoscopic) 1/NR/1/1  RYGBP (open & laparoscopic), sleeve gastrectomy or LAGB 1/NR/1/1  RYGBP (open) or gastroplasty 1/NR/1/1  Adverse outcome (death, nonroutine hospital transfer, one or more postoperative complications)  RYGBP (open & laparoscopic) 1/NR/1/1  Occurrence of composite event (defined as occurrence of any one of: death, VTE, PE, reoperation,  nondischarge at 30 days, repeat hospitalization within 30-days postdischarge)  RYGBP (open & laparoscopic) 1/NR/1/1  Readmission within 30 days of discharge  RYGBP (open & laparoscopic), gastroplasty (open & laparoscopic) 1/NR/1/1  Major morbidity (not specified)  RYGBP (open & laparoscopic) or BDP with DS 1/NR/0/1  Length of stay 2/NR/2/2  RYGBP (open) 1/NR/1/1  RYGBP (open & laparoscopic) 1/NR/1/1 | |
| Padwal  et al. 2011 [[41](#_ENREF_41)] | NR | Mortality 6/NR/5/6  Roux-en-Y gastric bypass 2/NR/2/2  Any bariatric treatment 3/NR/2/3  Gastric bypass 1/NR/1/1  Surgical Sequelae 6/NR/5/6  Roux-en-Y gastric bypass 1/NR/1/1  Any bariatric treatment 2/NR/1/2  Gastric bypass 1/NR/1/1  Adjustable gastric banding 1/NR/1/1  Gastric banding or gastroplasty 1/NR/?*/1  *significant for <100 vs. >100 and <150 vs. >150 cases per year but not for <25 vs. >25 and <50 vs. >50 cases per year | |
| Klarenbach et al. 2010 [[37](#_ENREF_37)] | NR | All-cause mortality 4/NR/3/4  Any bariatric treatment 3/NR/2/3  Gastric bypass 1/NR/1/1  Readmission  Gastric banding or gastroplasty 1/NR/?/1  Surgical sequelae 5/NR/4/5  Any bariatric treatment 2/NR/1/2  Gastric bypass 1/NR/1/1  Adjustable gastric banding 1/NR/1/1  Gastric banding or gastroplasty 1/NR/1/1  50% or more excess weight loss  Adjustable gastric banding (1) OR 1.7 (1.17 – 1.96)  Length of stay 2/NR/1/2  Roux-en-Y gastric bypass 1/NR/0/1  Any bariatric treatment 1/NR/1/1 | |
| **AAA** | | | |
| Young  et al. 2007 [[53](#_ENREF_53)] | No risk adjustment for all studies | Mortality 13/NR/12/14  (6) OR 0.56 (0.54 – 0.57), I^2^=23.7% (results derived by fixed effects model) | |
| Wilt  et al. 2006 [[50](#_ENREF_50)] | Age, sex | In-hospital mortality  Open surgical repair 4/NR/4/4 | |
| **Esophageal cancer** | | | |
| Brusselaers  et al. 2014 [[26](#_ENREF_26)] | NR | Long-term survival  (3) HR 1.14 (0.98 – 1.35), I^2^=0%; 3/NR/0/3 (without adjustment for hospital volume)  (2) HR 1.10 (1.02 – 1.18), I^2^=0%; 2/NR/1/2 (with adjustment for hospital volume) | |
| Wouters  et al. 2012 [[52](#_ENREF_52)] | Demographic data, comorbidities, and tumor characteristics for meta-analysis for survival (only demographic data for all survival studies)  Demographic data and comorbidities for meta-analysis for mortality (none for all mortality studies) | Survival NR/NR/2/4  (2) HR 1.16 (0.94 – 1.45), I^2^=48% (only high quality studies were considered for meta analysis)  Postoperative mortality 5/NR/5/9  (3) OR 0.87 (0.36 - 1.14), I^2^=75% (only high quality studies were considered for meta analysis)  Morbidity NR/NR/1/4  Quality of Life NR/NR/0/1 | |
| **Radical prostatectomy** | | | |
| Trinh  et al. 2013 [[47](#_ENREF_47)] | Age and CCI for perioperative complications  Age, CCI, marital status, education, income, population density, clinical stage, and tumor grade for postoperative complications  Age, CCI, and clinical stage for late urinary complications and long-term incontinence; PSA and clinical stage for positive surgical margins  Age, CCI and race for strictures  Clinical stage for biochemical recurrence  Age for blood transfusion  Age and CCI for risk of secondary therapy  Age, CCI, race, marital status, education, income, population density, clinical stage, and tumor grade for risk of adjuvant therapy  Age, CCI, race, hospital region, and surgical approach for rate of pelvic lymph node dissection | 30 day mortality  Open procedure NR/NR/0/1  Perioperative complications 4/NR/3/5  Open procedure 2/NR/2/3  Laparoscopic 2/NR/1/2  Complications (not specified)  Laparoscopic 1/NR/1/1  Late urinary complications  Open procedure 3(?)/NR/3(?)/3  Long-term incontinence  Open procedure 2/NR/2/2  (Anastomotic) strictures 4/NR/3/4  Open procedure 2/NR/2/2  Laparoscopic 2/NR/1/2  Estimated blood loss  Open procedure NR/NR/0/1  Blood transfusion 5/NR/3/5  Open procedure 3(?)/NR/1/3  Laparoscopic 2/NR/2/2  Risk of salvage therapy  Laparoscopic (1) OR 0.92 (0.88 – 0.98)  Rate of pelvic lymph node dissection  Open procedure 2/NR/2/2  Prolonged length of stay 8(?)/NR/7(?)/8  Open procedure 5(?)/NR/5(?)/5  Laparoscopic 3/NR/2/3 | Perioperative mortality  Open procedure (1) OR 0.32 (0.13 – 0.75)  Postoperative complications 1/NR/1/2  Open procedure 1/NR/1/1  Laparoscopic 0/NR/0/1  Recurrence-free survival time  Open procedure 1/NR/0/1  One year erectile and urinary complications, probability of recovery  Open procedure 1/NR/1/1  Positive surgical margins 5/NR/5/5  Open procedure 4/NR/4/4  Laparoscopic 1/NR/1/1  Biochemical recurrence 4/NR/4/5  Open procedure 3/NR/3/4  Laparoscopic 1/absolute risk difference 8%; 95% CI: 4-12%/1/1  Lymph node invasion  Open procedure (1) OR 1.8 (CI: NR)  Risk of secondary therapy 3/NR/2/3  Open procedure 2/NR/1/2  Laparoscopic 1/NR/1/1  Risk of adjuvant therapy 3/NR/3/3  Open procedure 1/NR/1/1  Laparoscopic 1/NR/1/1  Open + laparoscopic procedure 1/NR/1/1 |
| Wilt  et al. 2008 [[51](#_ENREF_51)] | Age for mortality  Age for complications | Surgery-related mortality ?/NR/?/3  (2) surgery related dose response per 10 procedures 0.02 (-0.01 – 0.4) % (heterogeneity not reported)  Complications NR/NR/NR/5  (4) surgery related dose response per 10 procedures -1.92 (-3.13 - -0.7) % (significant heterogeneity)  Length of stay days  (4) 1^st^ quartile vs. 4^th^ quartile -3.3 (-6.1 - -0.5) (heterogeneity not reported)  Long-term incontinence  (2) symptom dose response per 10 procedures -1.2 (-2.5 - -0.1)% (heterogeneity not reported)  Late urinary complication  (2) symptom dose response per 10 procedures -2.4 (-5 - -0.1) % (heterogeneity not reported) | |
| **Total knee arthroplasty** | | | |
| Lau  et al. 2012 [[38](#_ENREF_38)] | Age, comorbidity, gender, diagnosis, and HPV for 90-day mortality  Age, comorbidity, gender, diagnosis, and HPV for revision  Age, comorbidity, gender, diagnosis, and HPV for medical complications  Age, comorbidity, and gender for length of stay | 90-day mortality 3(?)/NR/0/3  In-hospital 30-day mortality 1/NR/NR/1  Infection 3/NR/3(?)/4  Revision 2/NR/0/2  Medical complications 0(?)/NR/0/3  Pneumonia (1) OR 0.72 (0.54 – 0.95)  Myocardial infarction (1) OR 0.90 (0.64 – 1.28)  Pulmonary embolus 1(?)/NR/0/1  WOMAC score 1/NR/1/1  Inability to flex the knee to 90 degrees (1) OR 0.34 (0.18 – 0.63)  Inability to achieve full extension at two years post operation (1) OR 0.45 (0.23 – 0.91)  Dissatisfied with total knee arthroplasty (1) OR 0.71 (0.30 – 1.67)  Procedure duration 1/NR/1/1  Readmission for surgery (1) OR 0.81 (0.41 – 1.62)  Transfusion rate 1/NR/1/1  Length of stay 4(?)/NR/4(?)/4  Solely results from tables were extracted due to contradictions in comparison with the text. | |
| Stengel  et al. 2004 [[45](#_ENREF_45)] | Age and sex for hospital mortality  Age and sex for deep venous thrombosis | Hospital mortality 2/NR/0/2  Mortality after 90 days 1/NR/0/1  Deep venous thrombosis 1/NR/0/2  Infection 1/NR/1/1  Readmission because of infection < 1 year 0/NR/0/1  Readmission because of infection < 3 year 0/NR/0/1  Pulmonary embolism 1/NR/0/1  Average length of stay 1/NR/1/1  Readmission because of revision < 1 year 0/NR/0/1  Readmission because of revision < 3 year 1/NR/0/1  All hospital complications 1/NR/?/1 | |
| **Breast cancer** | | | |
| Gooiker et al. 2010 [[30](#_ENREF_30)] | Age, sex, plus co-morbidity, and / or severity | Survival 7/NR/6/7  (4, 3) HR 1.22 (1.08 – 1.39), I^2^=59%; RR 1.18 (1.10 – 1.25), I^2^=0.0% | |
| **CABG** | | | |
| **Sepehripour**  **et al. 2013 [**[**44**](#_ENREF_44)**]** | **NR** | **(Risk-adjusted) mortality 1/NR/1/1**  **In-hospital mortality 1/NR/0/2**  **Stroke 1/NR/0/1**  **Postoperative myocardial infarction 1/NR/1/1**  **Renal failure 1/NR/0/1**  **Conversion to ONCAB (not off-pump) 1/NR/0/1**  **Grafts per patient 1/NR/1/1**  **Complete revascularization 1/NR/1/1** | |
| **Cystectomy** | | | |
| Goossens-Laan et al. 2011 [[32](#_ENREF_32)] | Age, sex, comorbidity for mortality | Survival  (1) HR 1.20 (0.88 – 1.67)  Postoperative Mortality 2/NR/2/2  (2) OR 0.58 (0.46 – 0.73), I^2^=50% | |
| **Head & neck cancer** | | | |
| Eskander et al. 2014 [[28](#_ENREF_28)] | NR | 5-year overall survival  Oral cavity resection (1) HR 1.23 (1.12 – 1.36)  3-year overall survival  Oral cavity with flap or predicted reconstruction (1) HR 1.52 (1.12 – 2.07)  Long-term mortality (3 or 5 years) 2/NR/2/2  Oral cavity (2) HR 0.77 (0.64 – 0.92), I^2^=0%  In-hospital mortality  Larynx 1/NR/0/2  Oropharingeal 1/NR/0/2  Regional recurrence after at least nine months of follow-up  Neck dissection 1/NR/1/1  Number of lymph nodes from neck dissection  Neck dissection 1/NR/1/1  Length of stay  Layrynx 1/NR/1/2  Oropharyngeal 0/NR/0/2  Oral cavity with flap reconstruction 1/NR/1/2 | |
| **Lung cancer** | | | |
| Van Meyenfeldt et al. 2012 [[49](#_ENREF_49)] | Age, sex, and comorbidities | Postoperative mortality 2/NR/2/2  (2) OR 0.677 (0.424 – 1.079), I^2^=66% | |
| **Norwood procedure** | | | |
| Pieper et al. 2014 [[42](#_ENREF_42)] |  | Survival 1/NR/0/1  Mortality 2/NR/1/2  Renal failure (1) OR 3.22 (0.92 – 11.11)  Time to first extubation 1/NR/0/1  Length of ventilation 1/NR/0/1 | |
| **Pancreatic surgery** | | | |
| Gooiker et al. 2011 [[31](#_ENREF_31)] | Age, sex, and comorbidities | Postoperative mortality 3/NR/2/3  (2, 1) OR 0.46 (0.17 – 1.26), I^2^=94%; HR 0.49 (0.29 – 0.84) | |
| **PCI** | | | |
| **Strom**  **et al. 2014 [**[**46**](#_ENREF_46)**]** | **NR (as data supplements with study characteristics are not available)** | **In-hospital and 30-day mortality 7/NR/1/13**  **(13) OR 0.96 (0.86 – 1.08), I^2^=61.4%**  **Major adverse cardiac events 7/NR/5/8**  **(8) OR 0.62 (0.40 – 0.97), I^2^=96.6%** | |
| **Trauma** | | | |
| Caputo et al. 2014 [[27](#_ENREF_27)] | NR | In-hospital mortality  1/NR/NR/4 | |
| **Several procedures / conditions** | | | |
| Gruen et al. 2009 [[3](#_ENREF_3)] | Age, sex, and comorbidity for esophagus  Age, sex, comorbidity, and stage of disease for stomach  Age, sex, comorbidity, stage of disease, and hospital volume for colon  Age, sex, and stage of disease for esophagus  Age, sex, and type of procedure for pancreas  Age, sex, and stage of disease for colon and rectum | Mortality (short-term)  Esophagus NR/NR/6/6  Stomach NR/NR/2/4  Pancreas NR/NR/5/11  Colon NR/NR/5/6  Rectum NR/NR/0/4  Colon and rectum (no specification) NR/NR/2/7  Mortality (long-term)  Esophagus NR/NR/0/2  Stomach NR/NR/0/2  Pancreas NR/NR/1/2  Colon NR/NR/1/1  Rectum NR/NR/2/4  Colon and rectum (no specification) NR/NR/2/5 | |
| Miyata et al. 2007 [[40](#_ENREF_40)] | NA | Mortality  Total gastrectomy 1/NR/0/1  Pancreatic leakage 1/NR/0/1  Complications  Total gastrectomy 1/NR/1/1 | |
| Gandjour et al. 2003 [[29](#_ENREF_29)] | NR | Mortality  Pediatric heart surgery 1/NR/1/1  Coronary stent placement 1/NR/1/1  Carotid endarterectomy 5/NR/2/5  Surgery for ruptured abdominal aortic aneurysm 1/NR/1/1  Surgery for unruptured abdominal aortic aneurysm 1/NR/1/1  Coronary artery bypass grafting 1/NR/0/1  Percutaneous transluminal coronary angioplasty 5/NR/2/6  Surgery for leg ischemia 2/NR/0/2  Surgery for rectal cancer 1/NR/1/1  Surgery for colorectal cancer 3/NR/1/3  Pancreatectomy 1/NR/1/1  Surgery for lung cancer 1/NR/0/1  Primary total hip and knee replacement 1/NR/1/1  Revision total hip and knee replacement 1/NR/1/1  Total hip arthroplasty 2/NR/0/2 | |
| Halm et al. 2002 [[33](#_ENREF_33)] | NR | Hospital or 30-day mortality  Coronary angioplasty NR/0.06 (-0.3 – 0.7)*/1/5  Coronary artery bypass NR/2.2 (0.8 – 5.7)*/3/3  Pediatric cardiac surgery 1/2.9*/1/1  Carotid endarterectomy NR/1.4 (1.2 – 5.8)*/7/12  Surgery for unruptured abdominal aortic aneurysm NR/3.2*/0/1  Surgery for ruptured abdominal aortic aneurysm NR/14.5*#/3/3  Pancreatic cancer surgery NR/-(7.0 – 10.2)*/1/2  Colorectal cancer surgery NR/1.9 (1.4 – 1.9)*/4/5  Lung cancer surgery NR/1.1*/0/1  Gastric cancer surgery NR/-(4.0 – 5.7)*/2/2  Total hip replacement NR/-(0.0 – 1.7)*/2/3  5-year survival  Breast cancer 1/OR 1.18/1/1    * Median absolute difference in mortality rate for high vs. low volume (range)  # “value given only for one study, data from other studies were not available” | |
| Hillner et al. 2000 [[34](#_ENREF_34)] | NR | Not specified outcome  Pancreas 1/NR/NR/NR  Colorectal 2-4/NR/NR/NR  Breast 2-4/NR/NR/NR  Other gastrointestinal surgery -/-/-/0  Lung -/-/-/0  Ovary -/-/-/0  Prostate -/-/-/0  Lymphoma -/-/-/0  Testes -/-/-/0 | |
| **Several procedures / conditions for children** | | | |
| McAteer et al. 2013 [[39](#_ENREF_39)] | Disease severity for neurosurgery  Disease severity for urology | In-hospital mortality  Cardiac surgery 1/NR/1/1  Primary outcome (not specified)  Neurosurgery 3/NR/3/3  Urology (ureteral reimplantation) 2/NR/2/2 (positive association between volume and outcome in total)  Appendectomy NR/NR/NR/NR (positive association between volume and outcome in total)  Rate of complications  Otolaryngology (cleft lip repair) 1/NR/1/1 | |

BDP – Biliopancreatic diversion

BMI – Body mass index

CCI – Charlson comorbidity index

DS – Duodenal switch

HPV – Hospital procedure volume

HVS – High volume surgeons

LAGB – Laparoscopic adjustable gastric band

LOS – Length of stay

ME – Medicaid eligibility

NA – Not applicable

NR – Not reported

ONCAB – On-pump coronary artery bypass

OSA – Obstructive sleep apnoea

PE – Pulmonary embolism

PSA – Prostate-specific antigen

RYGBD – Roux-en-y gastric bypass

TO – Type of operation

VTE – Venous thromboembolism

WOMAC – Western Ontario and McMaster Universities Arthritis Index
